# Profilin1 is required for prevention of mitotic catastrophe in murine and human glomerular diseases

**DOI:** 10.1172/JCI171237

**Published:** 2023-12-15

**Authors:** Xuefei Tian, Christopher E. Pedigo, Ke Li, Xiaotao Ma, Patricia Bunda, John Pell, Angela Lek, Jianlei Gu, Yan Zhang, Paulina X. Medina Rangel, Wei Li, Eike Schwartze, Soichiro Nagata, Gabriel Lerner, Sudhir Perincheri, Anupama Priyadarshini, Hongyu Zhao, Monkol Lek, Madhav C. Menon, Rongguo Fu, Shuta Ishibe

**Affiliations:** 1 Section of Nephrology, Department of Internal Medicine, Yale School of Medicine, New Haven, Connecticut, USA.; 2 Department of Nephrology, Second Affiliated Hospital of Xi’an Jiaotong University, Xi’an, Shaanxi, China.; 3 Department of Genetics and; 4 Department of Biostatistics, Yale School of Public Health, New Haven, Connecticut, USA.; 5 Bioinformation Department, Suzhou SITRI Institute of Immunology Co. Ltd., Suzhou, Jiangsu, China.; 6 Departments of Surgical Pathology and Laboratory Medicine, Yale School of Medicine, New Haven, Connecticut, USA.

**Keywords:** Cell Biology, Nephrology, Cell cycle

## Abstract

The progression of proteinuric kidney diseases is associated with podocyte loss, but the mechanisms underlying this process remain unclear. Podocytes reenter the cell cycle to repair double-stranded DNA breaks. However, unsuccessful repair can result in podocytes crossing the G_1_/S checkpoint and undergoing abortive cytokinesis. In this study, we identified *Pfn1* as indispensable in maintaining glomerular integrity — its tissue-specific loss in mouse podocytes resulted in severe proteinuria and kidney failure. Our results suggest that this phenotype is due to podocyte mitotic catastrophe (MC), characterized histologically and ultrastructurally by abundant multinucleated cells, irregular nuclei, and mitotic spindles. Podocyte cell cycle reentry was identified using FUCCI2aR mice, and we observed altered expression of cell-cycle associated proteins, such as p21, p53, cyclin B1, and cyclin D1. Podocyte-specific translating ribosome affinity purification and RNA-Seq revealed the downregulation of ribosomal RNA-processing 8 (*Rrp8*). Overexpression of *Rrp8* in *Pfn1-*KO podocytes partially rescued the phenotype in vitro. Clinical and ultrastructural tomographic analysis of patients with diverse proteinuric kidney diseases further validated the presence of MC podocytes and reduction in podocyte PFN1 expression within kidney tissues. These results suggest that profilin1 is essential in regulating the podocyte cell cycle and its disruption leads to MC and subsequent podocyte loss.

## Graphical abstract

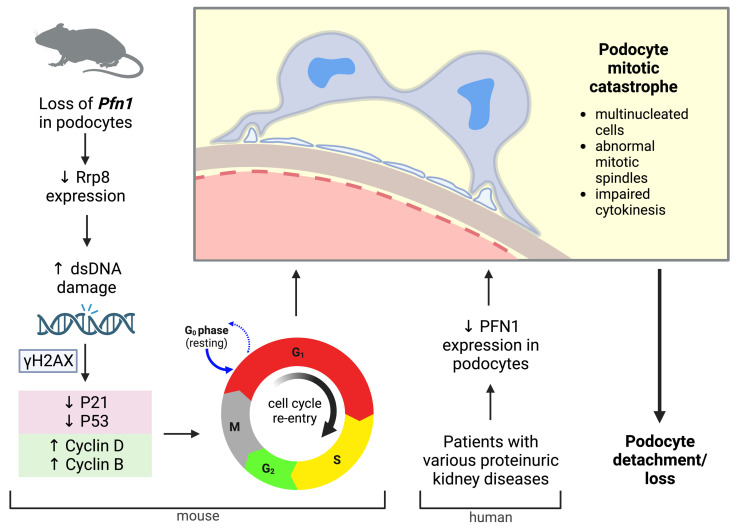

## Introduction

Podocytes are specialized terminally differentiated epithelial cells that line the kidney filtration barrier. Unlike typical epithelial cells that are compressed against their basement membrane, podocytes are situated on the urinary side, rendering them highly susceptible to the 180 liters of blood flow that gets filtered on a daily basis (1, 2). Detached podocytes are frequently observed in human urine following glomerular injury (3, 4). Due to their limited regenerative capacity, this irreplaceable loss results in damage to the glomerular filtration barrier and protein leakage and, often, culminates in kidney failure. Yet, the mechanism of how podocytes are lost remains elusive, as reports propose diverse processes, including apoptosis, necrosis, autophagy, pyroptosis, senescence, and anoikis (5, 6). Recent studies in human podocytes suggest that mitotic catastrophe (MC), characterized by abnormal chromosomal segregation occurring following DNA damage and manifesting histologically as podocyte multinucleation, may also play a vital role in how podocytes are lost following injury (7–11). Injured podocytes reenter the cell cycle, and during the G_1_ phase, they engage in protein synthesis and augment organelle numbers, leading to hypertrophy (12). Yet, in cases in which DNA damage is severe and irreparable, asymmetric chromosomal division gives rise to MC, leading to podocyte loss following injury.

We and other researchers have previously shown that an intact podocyte actin cytoskeleton plays a profound role in maintaining the integrity of the glomerular filtration barrier (1, 13–16). While pursuing understanding of key proteins in the process, we investigated the role of profilin1, which has been demonstrated to be critical in inhibiting actin filament polymerization by sequestering G actin (14, 17). Profilin1 has also been known to interact with Ena/vasodilator-stimulated phosphoprotein (VAPS), formins, and Arp2/3-dependent Wiskott-Aldrich syndrome protein (WASP) (18, 19). To further determine the impact of *Pfn1* in podocytes, we generated podocyte-specific *Pfn1*-KO mice, which revealed severe proteinuria, glomerulosclerosis, and kidney failure. Unexpectedly, although no apparent differences were observed in phalloidin staining of *Pfn1*-KO podocytes, we observed the presence of multiple nuclei in mutant podocytes both in vitro and in vivo. Furthermore, our investigations extended to human proteinuric kidney diseases, such as focal segmental glomerulosclerosis (FSGS), diabetic kidney disease (DKD), primary membranous nephropathy (pMN), lupus nephritis (LN), and IgA nephropathy (IgAN). Through transmission electron microscopy (TEM), we identified abundant podocytes with multiple nuclei and observed a reduction in profilin1 immunoreactivity. As recent evidence suggests that profilin1 may serve as an important regulator of cell cycle progression (20), we studied its effect on this process by using fluorescence-ubiquitination-based cell cycle indicator (FUCCI2aR) mice, which allow for in vivo podocyte-specific interrogation of cell cycle status using immunofluorescent labeling (12, 21). Additionally, we performed a podocyte-specific translating ribosome affinity purification (TRAP) RNA-Seq to conduct an unbiased screen on control and mutant podocytes to further elucidate the underlying mechanisms (22). We observed that *Pfn1*-KO podocytes had entered the cell cycle in the G_1_, S, and G_2_ phases and this regulation was partially mediated by a reduction in ribosomal RNA-processing 8 (*Rrp8*), a gene known to be involved in the energy-dependent silencing of ribosomal DNA, histone recruitment, and DNA repair (23, 24). Together, our results suggest that profilin1 plays a prominent role in podocyte cell cycle regulation and that disruption of this protein contributes to podocyte loss by inducing MC.

## Results

### Loss of podocyte-associated Pfn1 results in severe albuminuria, kidney failure, and death.

**Figure 1:**
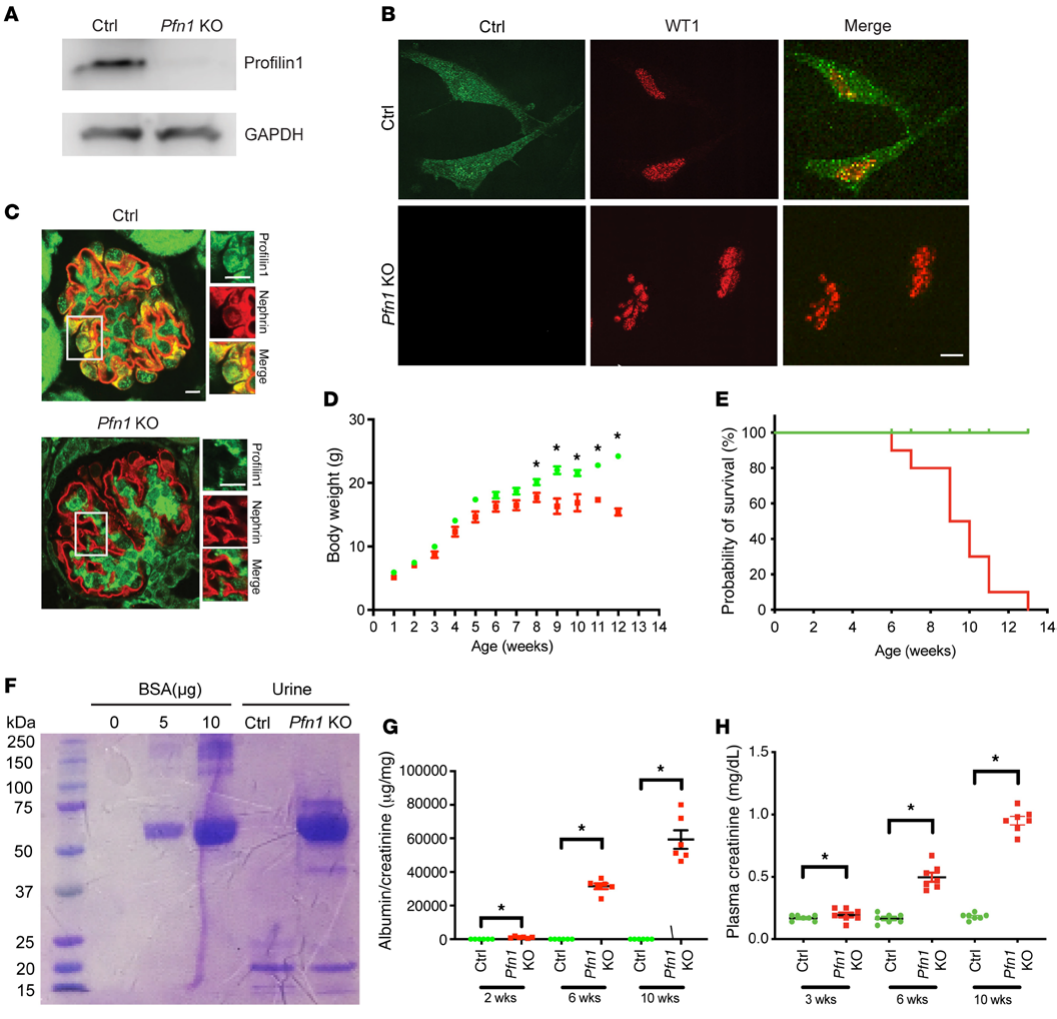
Generation of podocyte-specific *Pfn1-*KO mice results in severe proteinuria and kidney failure. (**A**) Representative immunoblot images of profilin1 expression in primary podocytes freshly isolated from littermate control (Ctrl) and *Pfn1*-KO mice (age P7). (**B**) Representative immunofluorescence images of profilin1 (green) and WT1 (red) in control and *Pfn1*-KO primary podocytes. Scale bar: 10 μm. (**C**) Representative immunofluorescence images of profilin1 (green) and nephrin (red) on kidney sections of control and *Pfn1-*KO mice (age 3 weeks). Scale bars: 10 μm. (**D**) *Pfn1-*KO mice (red) failed to gain body weight by 8 weeks of age compared with control mice (green). *n* = 9 mice. *P < 0.05 vs. control. (**E**) The survival curve of *Pfn1-*KO mice (red) demonstrates approximately 90% death by 12 weeks of age. *n* = 8 mice. (**F**) SDS-PAGE (Coomassie blue staining) of standard BSA and of urine samples from *Pfn1*-KO mice at 4 weeks of age demonstrates albuminuria. Equal volumes of standard BSA and urine (4 μL) were loaded in each lane. (**G**) Quantification of urine albumin normalized to creatinine at 2, 6, and 10 weeks of age. *n* = 6 mice. *P < 0.05 vs. control. (**H**) Elevated plasma creatinine in *Pfn1-*KO mice at 3, 6, and 10 weeks of age. *P < 0.05 vs. control. Statistics were analyzed via a 2-tailed *t* test.

To examine the importance of profilin1 in podocytes, we generated podocyte-specific *Pfn1*-KO mice through the mating of *Pfn1^fl/fl^* mice with the *Nphs2(Pod)-Cre Rosa-DTR^fl^* mice (*Pfn1*-KO mice). To ensure a pure population of primary podocytes, we utilized the *loxP* sites flanking the diphtheria toxin receptor (DTR) (12, 25) in combination with a podocyte-specific Cre recombinase promoter (*Pod-Cre*; podocin). This strategy spared podocytes from cell death upon diphtheria toxin administration while eliminating all non-*Pod*-*Cre*–expressing kidney cells. *Pfn1*-KO mice were born at the expected Mendelian frequency, and the mutant mice were verified by tail genotyping for the appropriate genes (Supplemental Figure 1A; supplemental material available online with this article; https://doi.org/10.1172/JCI171237DS1). Loss of podocyte *Pfn1* expression in the mutant mice was further confirmed by real-time PCR (Supplemental Figure 1B), Western blotting (Figure 1A), and immunofluorescence (Figure 1B), using isolated primary podocyte lysates. Immunofluorescence of kidney sections (Figure 1C) also demonstrated in vivo loss of profilin1 expression in podocytes. *Pfn1*-KO mice appeared normal at birth but failed to gain body weight at 8 weeks of age, compared with their littermate controls (Figure 1D), and approximately 90%–100% of mice died by 13 weeks of age (Figure 1E). Urinalysis on *Pfn1*-KO mice using SDS-PAGE followed by Coomassie Blue staining demonstrated marked albuminuria (Figure 1F), which progressed from postnatal week 2 and was quantified by the albumin/creatinine ratio using ELISA (Figure 1G). Finally, *Pfn1*-KO mice exhibited biochemical evidence of kidney failure with elevated plasma creatinine when compared with littermate controls (Figure 1H).

### Loss of podocyte-specific Pfn1 results in glomerulosclerosis, tubulointerstitial injury, and foot process effacement.

**Figure 2:**
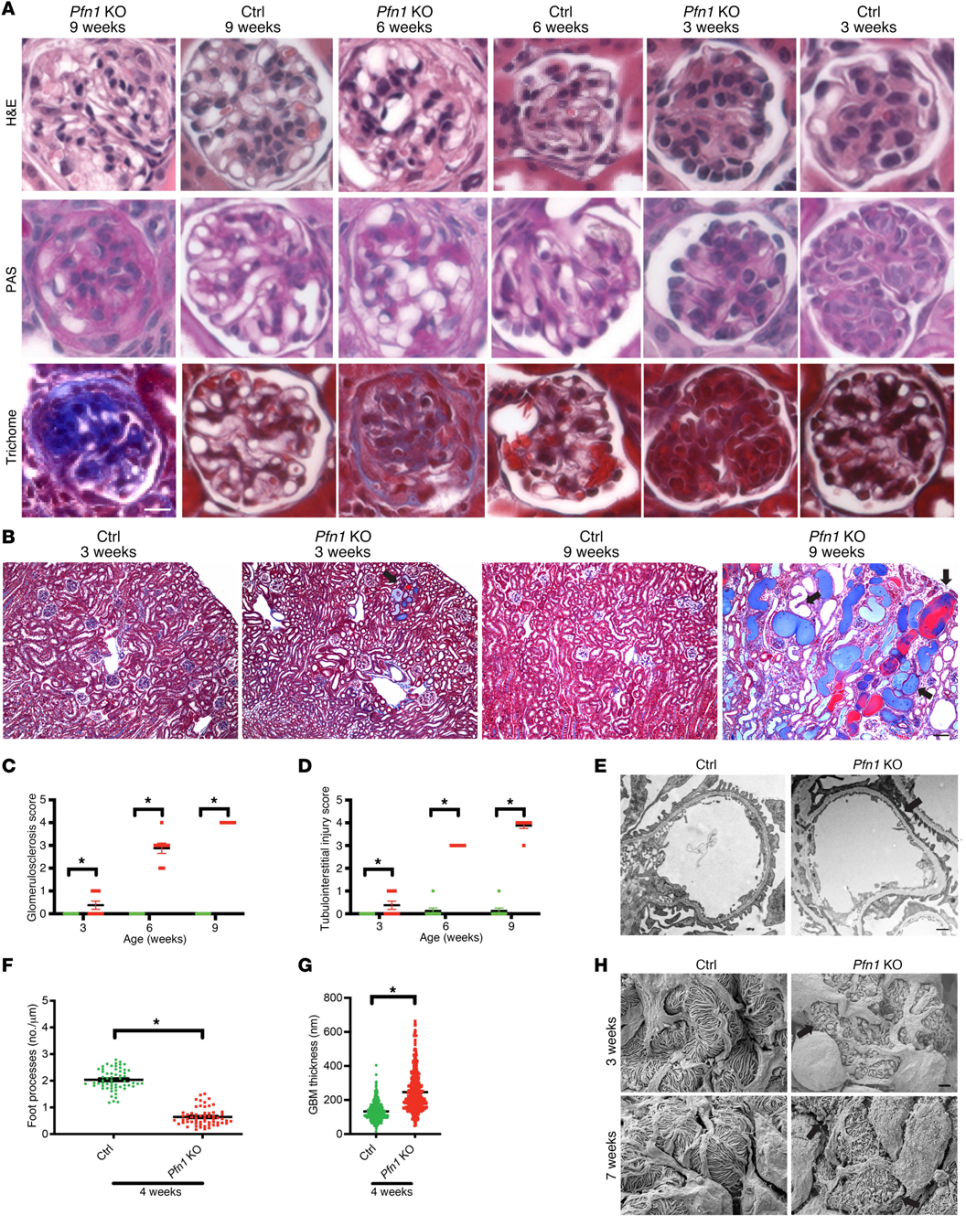
Loss of podocyte *Pfn1* results in progressive glomerulosclerosis and tubulointerstitial injury. (**A**) Representative light microscopy images (H&E, periodic acid–Schiff [PAS], and Masson’s trichrome) of control and *Pfn1-*KO mouse glomeruli at 3, 6, and 9 weeks of age. Scale bar: 25 μm. (**B**) Representative Masson’s trichrome staining in control and *Pfn1-*KO mouse kidneys at 3 and 9 weeks of age. Arrowheads depict dilated tubules, proteinaceous casts, and interstitial fibrosis. Scale bar: 100 μm. (**C**) Quantification of glomerulosclerosis in **A**. *n* = 8 mice. *P < 0.05 vs. control. (**D**) Quantification of tubulointerstitial injuries in **B**. *n* = 8 mice. *P < 0.05 vs. control. (**E**) Representative transmission electron micrography (TEM) of foot processes in control and *Pfn1*-KO mice at 4 weeks of age. Arrowheads depict podocyte foot process effacement. Scale bar: 1 μm. (**F**) Quantification of the number of foot processes per micrometer of GBM in **E**. *n* = 4 mice, *P < 0.05 vs. control. (**G**) Quantification of GBM thickness in **E**. *n* = 4 mice. *P < 0.05 vs. control. (**H**) Representative scanning electron micrography illustrating the ultrastructure of the glomerulus of control and *Pfn1*-KO mice at 3 and 7 weeks of age. Arrowheads demonstrate loss of foot process interdigitations. Scale bar: 1 μm. Statistics were analyzed via a 2-tailed *t* test.

As the mutant mice had severe albuminuria and evidence of kidney failure, we sought to examine renal histopathologic changes. Histologic examination revealed progressive glomerulosclerosis starting at 3 weeks of age (Figure 2A, quantified in Figure 2C). Additionally, we observed tubulointerstitial injury, including tubular atrophy, tubular dilation, proteinaceous casts, and interstitial fibrosis (Figure 2B, quantified in Figure 2D) in the *Pfn1-*KO mouse kidneys. To gain further insights into the ultrastructural features of the glomerulus, we performed both TEM and scanning electron microscopy (SEM) on *Pfn1*-KO and control mouse kidneys. TEM demonstrated severe podocyte foot process effacement and increased glomerular basement membrane (GBM) thickness in the mutant mice when compared with control mice (Figure 2E, quantified for foot process effacement in Figure 2F and for GBM thickness in Figure 2G). SEM analysis further demonstrated a dramatic loss of podocyte foot process interdigitations and destruction of the major processes (Figure 2H).

### Loss of podocyte Pfn1 results in MC, chromosomal instability, and DNA damage.

**Figure 3:**
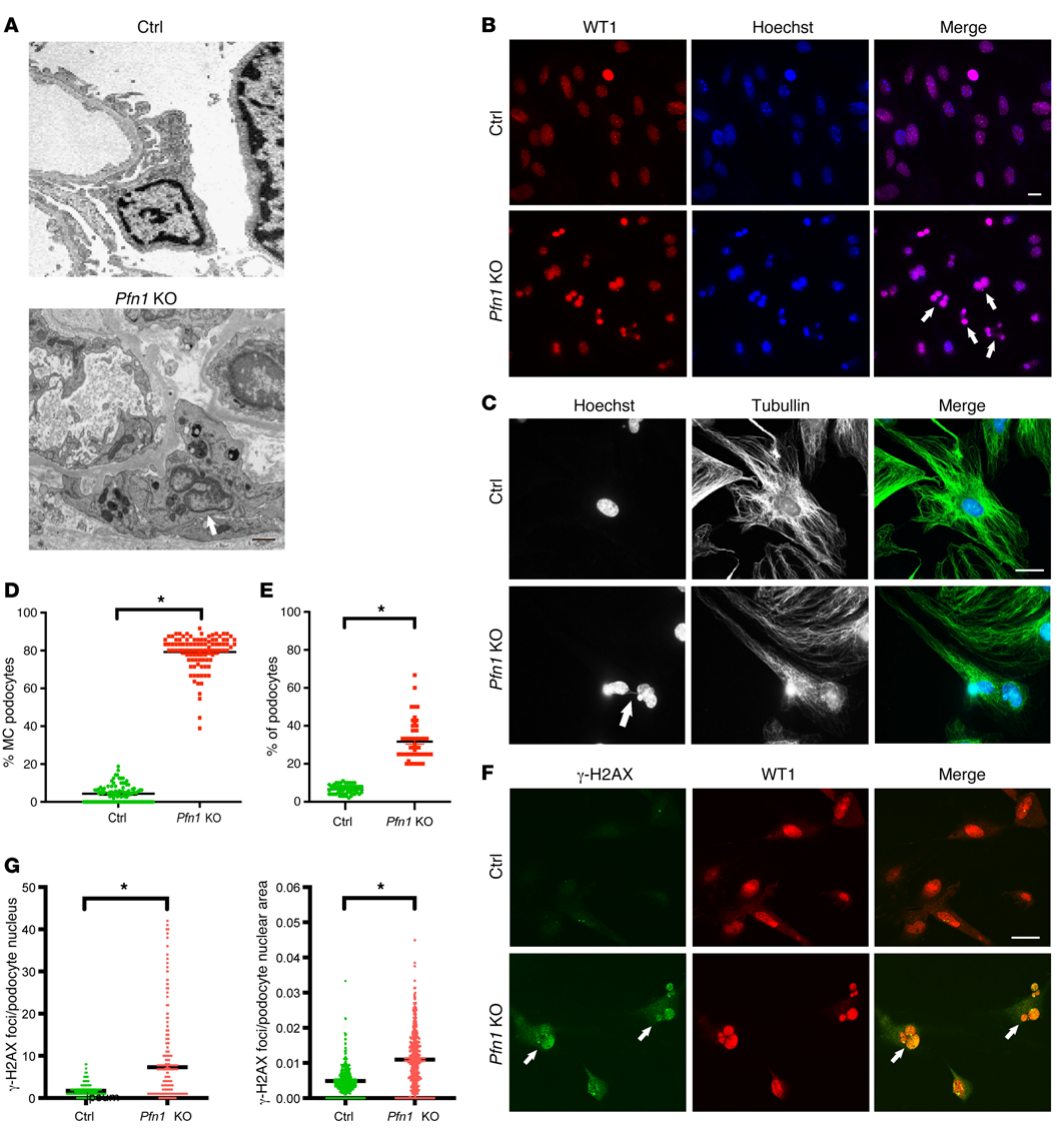
Loss of podocyte *Pfn1* results in morphologic MC appearance, chromosomal instability, and dsDNA damage. (**A**) Representative images of transmission electron micrography demonstrate abnormal MC podocytes in *Pfn1*-KO mice (arrow) compared with control glomeruli at 4 weeks of age. Scale bar: 1 μm. (**B**) Immunofluorescence images of primary podocytes isolated from control and *Pfn1*-KO mice at P7 stained with WT1 (red, podocyte marker) and Hoechst (blue, DNA marker) showing abnormal MC podocytes in *Pfn1-*KO mice (arrow) compared with control podocytes. Scale bar: 20 μm. (**C**) Immunofluorescence images of primary culture podocytes isolated from control and *Pfn1*-KO mice stained with Hoechst and anti-tubulin antibody showing the chromosome bridge (arrow) in a MC *Pfn1-*KO podocyte. Scale bar: 20 μm. (**D**) Quantification of the percentage of MC podocytes per field of view in **B**. Total of 100 fields of view in 5 independent experiments. (**E**) Quantification of the percentage of chromosome bridge per field of view in **C**. *n* = 5 independent experiments. *P < 0.05 vs. control. (**F**) Immunofluorescence images of primary culture podocytes stained with γ-H2AX (green, double-strand breaks [DSBs] marker) and WT1 (red) showing abnormal MC podocytes in *Pfn1-*KO mice, as indicated by the arrows. Scale bar: 20 μm. (**G**) Quantification of γH2AX foci per podocyte nucleus (left) and per podocyte nuclear area (right) in **F**. Total of 400 cells in 5 independent experiments. *P < 0.05 vs. control. Statistics were analyzed via a 2-tailed *t* test.

Close examination of podocyte ultrastructure by TEM demonstrated a striking presence of multinucleated podocytes in *Pfn1*-KO mice when compared with podocytes from control mice (Figure 3A). As multinucleation (mostly binucleation) is a prominent morphological feature of MC (26), these findings suggest a possible link between MC and the phenotype observed in the *Pfn1*-KO mice. Immunostaining of freshly isolated primary *Pfn1*-KO and littermate control podocytes, with podocyte-specific nuclear marker WT1 revealed a robust presence of MC podocytes in the mutant podocytes (Figure 3B, quantified in Figure 3D). To further validate these findings in human podocytes, we generated human *PFN1*-KO podocytes using CRISPR/Cas9 technology. Successful loss of human *PFN1* in podocytes was confirmed by real-time PCR (Supplemental Figure 2A) and Western blotting (Supplemental Figure 2B). Similarly, immunostaining with WT1 demonstrated numerous MC podocytes compared with the controls (Supplemental Figure 2C, quantified in 2D). As double-stranded DNA (dsDNA) damage has been shown to induce MC, leading to chromosomal loss and increased accumulation of missegregated chromosomal parts during mitosis (27), we stained for tubulin and Hoechst, a DNA marker. *Pfn1*-KO podocytes demonstrated abnormal mitotic spindles (Figure 3C, quantified in Figure 3E). Given that *Pfn1-*KO podocytes showed aneuploidy and chromosomal instability, we next determined whether the profilin1 deficiency results in dsDNA damage by staining with phosphorylated histone H2AX (γH2AX), a sensitive molecular marker of double-strand breaks (DSBs) (28). We used positive controls to confirm the expression of γH2AX in podocytes, wherein WT mouse podocytes were exposed to freshly prepared 0.5 μg/mL adriamycin for 12 hours (29, 30) or 20 μM hydrogen peroxide (H_2_O_2_) for 24 hours (31, 32). Following these treatments, we observed increased expression levels of γH2AX in the podocytes, with clear localization in the nucleus (Supplemental Figure 3A for adriamycin treatment and Supplemental Figure 3B for the H_2_O_2_ treatment). Immunofluorescence revealed increased podocyte nuclear γH2AX foci in both *Pfn1*-KO podocytes (Figure 3F, quantified in Figure 3G) and *PFN1*-KO podocytes (Supplemental Figure 2E). Finally, to further evaluate the fate of injured podocytes, we performed the TUNEL assay and stained for senescence-associated β-galactosidase (SA-β-gal) activity to assess for apoptosis or senescence, respectively (6). Kidney tissue of mutant mice at 4 weeks of age was devoid of TUNEL-positive podocytes but positivity was observed in kidney tubular cells (Supplemental Figure 4A, quantified in Supplemental Figure 4B). Furthermore, in *Pfn1*-KO primary podocytes and kidney tissues, SA-β-gal activity was not observed in the podocytes (Supplemental Figure 4C) (12) or the glomerulus (Supplemental Figure 4D). Considering profilin1’s known involvement in actin cytoskeleton regulation in other cell types (33, 34), we investigated phalloidin staining in the mutant podocytes. Unexpectedly, compared with controls, we did not observe a striking difference in the phalloidin pattern in *Pfn1*-KO primary podocytes (Supplemental Figure 5A) or human *PFN1*-KO podocytes (Supplemental Figure 5B). Similarly, there was no obvious difference in the tubulin pattern observed in *Pfn1*-KO primary podocytes (Supplemental Figure 5C) or human *PFN1*-KO podocytes (Supplemental Figure 5D). Subsequently, we examined the effects of profilin1 on podocyte spreading and migration. We observed a modest, though significant reduction in cell spreading and migration in both the primary podocytes from *Pfn1*-KO mice and human *PFN1*-KO podocytes compared with their respective controls (Supplemental Figure 6A, quantified in Supplemental Figure 6B for mouse *Pfn1* podocyte spreading assay; Supplemental Figure 6C, quantified in Supplemental Figure 6D for human *PFN1* podocyte spreading assay; Supplemental Figure 6E, quantified in Supplemental Figure 6F for mouse *Pfn1* podocyte migration assay; and Supplemental Figure 6G, quantified in Supplemental Figure 6H for human *PFN1* podocyte migration assay). Despite its known involvement in the actin cytoskeleton regulation in other cell types, profilin1’s effect on podocyte actin dynamics in vitro appears to be less evident than what is likely occurring in vivo where there is profound foot process effacement.

### Loss of podocyte Pfn1 results in DNA damage response.

**Figure 4:**
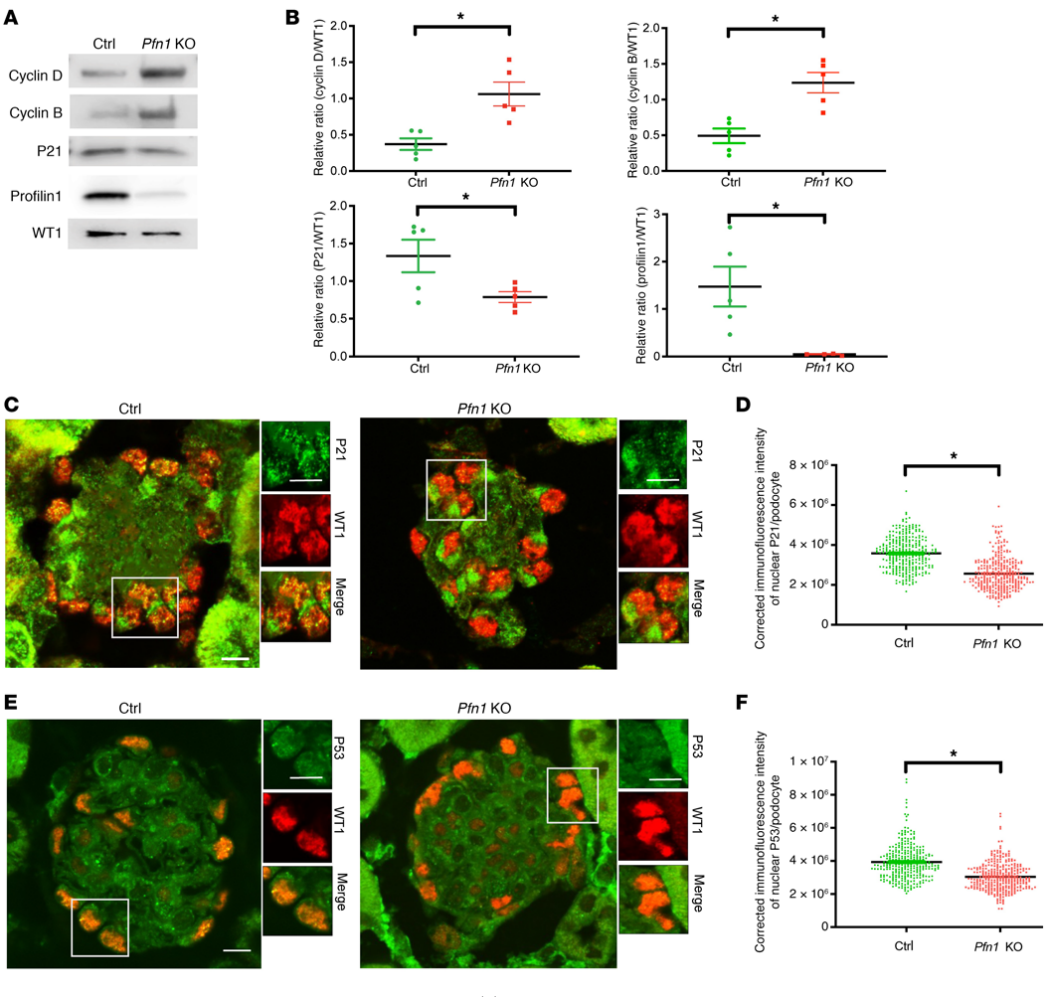
Loss of podocyte *Pfn1* activates the DNA damage response. (**A**) Representative immunoblot images of cyclin D1, cyclin B1, P21, profilin1, and WT1 as loading control in control and *Pfn1*-KO mouse primary podocytes. (**B**) Quantification of immunoblots in **A**. *n* = 5 independent experiments. (**C**) Representative immunofluorescence image of P21 expression in control and *Pfn1-*KO mouse glomeruli at 5 weeks of age stained with P21 (green) and WT1 (red). Scale bar: 20 μm. (**D**) Quantification of immunofluorescence intensity of nuclear P21 per podocyte in **C**. Total of 310 podocytes in 5 mice. (**E**) Representative immunofluorescence images of P53 in control and *Pfn1-*KO mouse glomeruli at 5 weeks of age stained with P53 (green) and WT1 (red). Scale bar: 20 μm. (**F**) Quantification of immunofluorescence intensity of nuclear P53 per podocyte in **E**. Total of 310 podocytes in 5 different mice. *P < 0.05 vs. control. Statistics were analyzed via a 2-tailed *t* test.

To next investigate whether dsDNA damage and aneuploidy induced by aberrant entrance into the cell cycle contributed to the pathophysiological process, we assessed key cell cycle regulatory proteins. In mouse *Pfn1-*KO podocytes, a marked increase in the cyclin D1 and cyclin B1 expression was observed, while there was a reduction in p21 expression (Figure 4A, quantified in Figure 4B) that was further recapitulated in the *PFN1*-KO podocytes (Supplemental Figure 2G, quantified in Supplemental Figure 2H). Furthermore, immunofluorescence staining of mouse kidney tissues revealed decreased podocyte p21 (Figure 4C, quantified in Figure 4D) and p53 (Figure 4E, quantified in Figure 4F) nuclear expression in *Pfn1-*KO mice when compared with littermate controls. These findings suggest that podocyte-specific *Pfn1* deletion results in abnormal cell cycle entrance.

### Deletion of Pfn1 in podocytes results in cell cycle entry.

**Figure 5:**
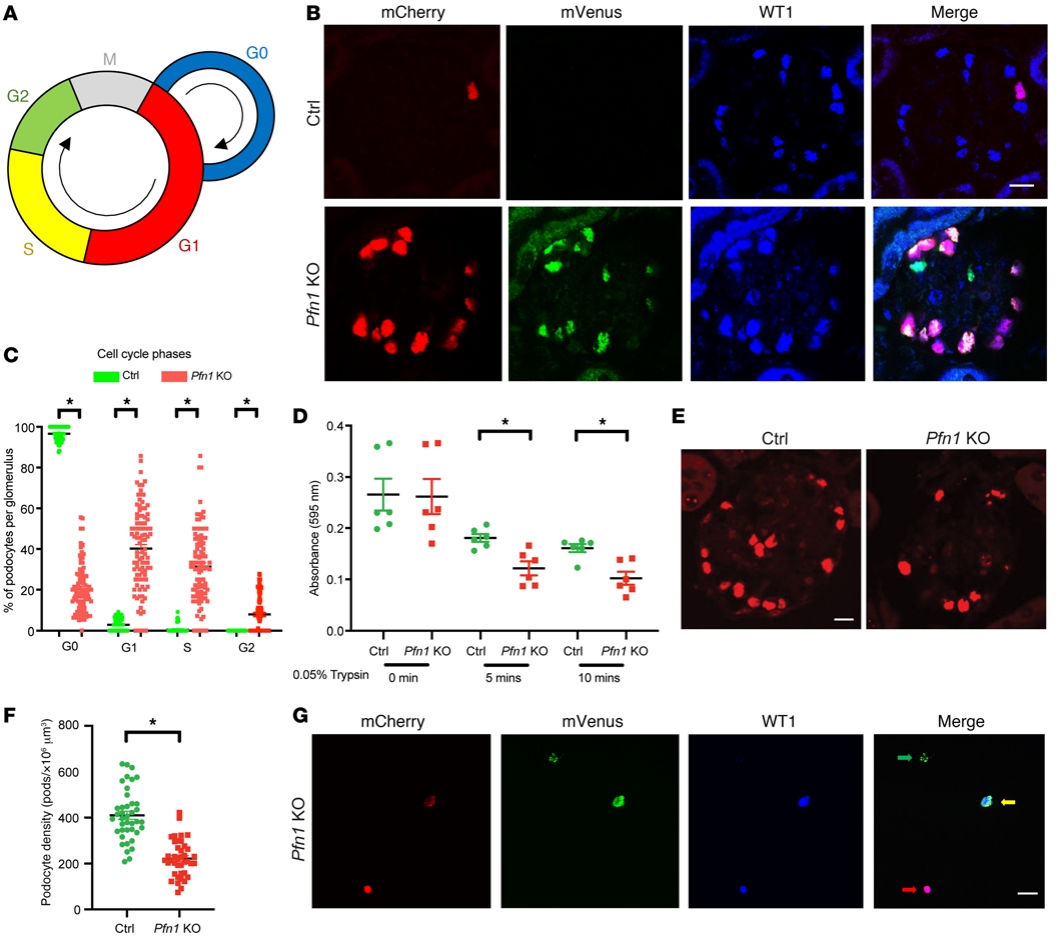
Loss of podocyte *Pfn1* results in podocytopenia and cell cycle entry. (**A**) A schematic representing cell cycle phase visualization by different colors in podocytes from the *R26Fucci2aR* *Pfn1*-KO mice. G1 phase nuclei (mCherry, red); S phase nuclei (both mCherry and mVenus, yellow); and G2 phase nuclei (mVenus, green). (**B**) Representative immunofluorescence images of podocytes in control*-FUCCI* and *Pfn1-*KO-*FUCCI* glomeruli at 4 weeks of age stained with Cherry (red), Venus (green), and WT1 (blue). Scale bar: 20 μm. (**C**) Quantification of the distribution of podocytes in cell cycle phase in **B**. Total of 100 glomeruli in 5 different mice. (**D**) Primary podocytes isolated from control and *Pfn1*-KO mice demonstrated a significant decrease in adhesion after plating for 5 minutes and 10 minutes on the collagen type I–coated plates. *n* = 6 independent experiments. (**E**) Representative immunofluorescence images of podocytes in control and *Pfn1-*KO glomeruli at 7 weeks of age stained with WT1 (red). Scale bar: 20 μm. (**F**) Quantification of podocyte density in glomeruli in **E**. Total of 40 glomeruli from 5 different mice. (**G**) Representative immunofluorescence images of urinary podocytes from control and *Pfn1*-KO mice at 4 weeks of age stained with mCherry (red), mVenus (green), and WT1 (blue). G_1_ phase podocyte nuclei (red arrow), S phase podocyte nuclei (yellow arrow), and G_2_ phase podocyte nuclei (green arrow) were depicted. Scale bar: 20 μm. *P < 0.05 vs. control. Statistics were analyzed via a 2-tailed *t* test.

To further investigate the cell cycle phase distribution in podocytes, we employed a mouse model by crossing the *R26Fucci2aR* mice with the *Pfn1^fl/+^ Pod-Cre RosaDTR^fl^* mice, generating the *Pfn1-*KO-*FUCCI* and control-*FUCCI* mice. The FUCCI2aR system leverages the fluorescent probes mCherry-Cdt1 and mVenus-geminin, which are involved in the regulation of cell cycle phases at G_1_ and G_2_, respectively (35). In this system, cells in the G_1_ phase emit a red nuclear fluorescence, while cells in the G_2_ phase emit a green nuclear fluorescence, and cells in the S phase exhibit yellow staining. Quiescent state G_0_ phase nuclei do not display any fluorescence (21) (Figure 5A). Examination of glomeruli from the control-*FUCCI* and *Pfn1-*KO*-FUCCI* mice at 4 weeks of age revealed an abundance of podocytes in G_1_, S, and G_2_ phases in the mutant mice (Figure 5B, quantification in Figure 5C). Additionally, cell cycle phase analysis of *Pfn1*-KO podocytes and control podocytes in vitro using flow cytometry displayed similar results (Supplemental Figure 7A). To determine whether the podocytopenia was due to reduced podocyte adhesion, crystal violet was used to identify control and *Pfn1*-KO podocytes after plating on collagen type I. *Pfn1*-KO podocytes showed decreased adhesion relative to control podocytes (Figure 5D), a finding further supported in human *PFN1*-KO podocytes (Supplemental Figure 2F). To next assess whether the alternation of cell cycle observed in the *Pfn1*-KO podocytes resulted in reduced podocyte number, we performed WT1 staining and revealed a marked loss of podocytes at 7 weeks of age (Figure 5E, quantified in Figure 5F). Accordingly, we observed *Pfn1-*KO*-FUCCI* mouse podocytes from collected urine samples in the G_1_ phase, S phase, and G_2_ phase at 4 weeks of age (Figure 5G). Collectively, these results suggest that loss of podocyte-associated *Pfn1* not only induces cell cycle reentry, but also “commits” cells to cellular mitosis, leading to the formation of MC podocytes that detach from the GBM, ultimately causing a reduction in podocyte number.

### Identification of Rrp8 involved in preventing podocyte loss through TRAP RNA-Seq.

**Figure 6:**
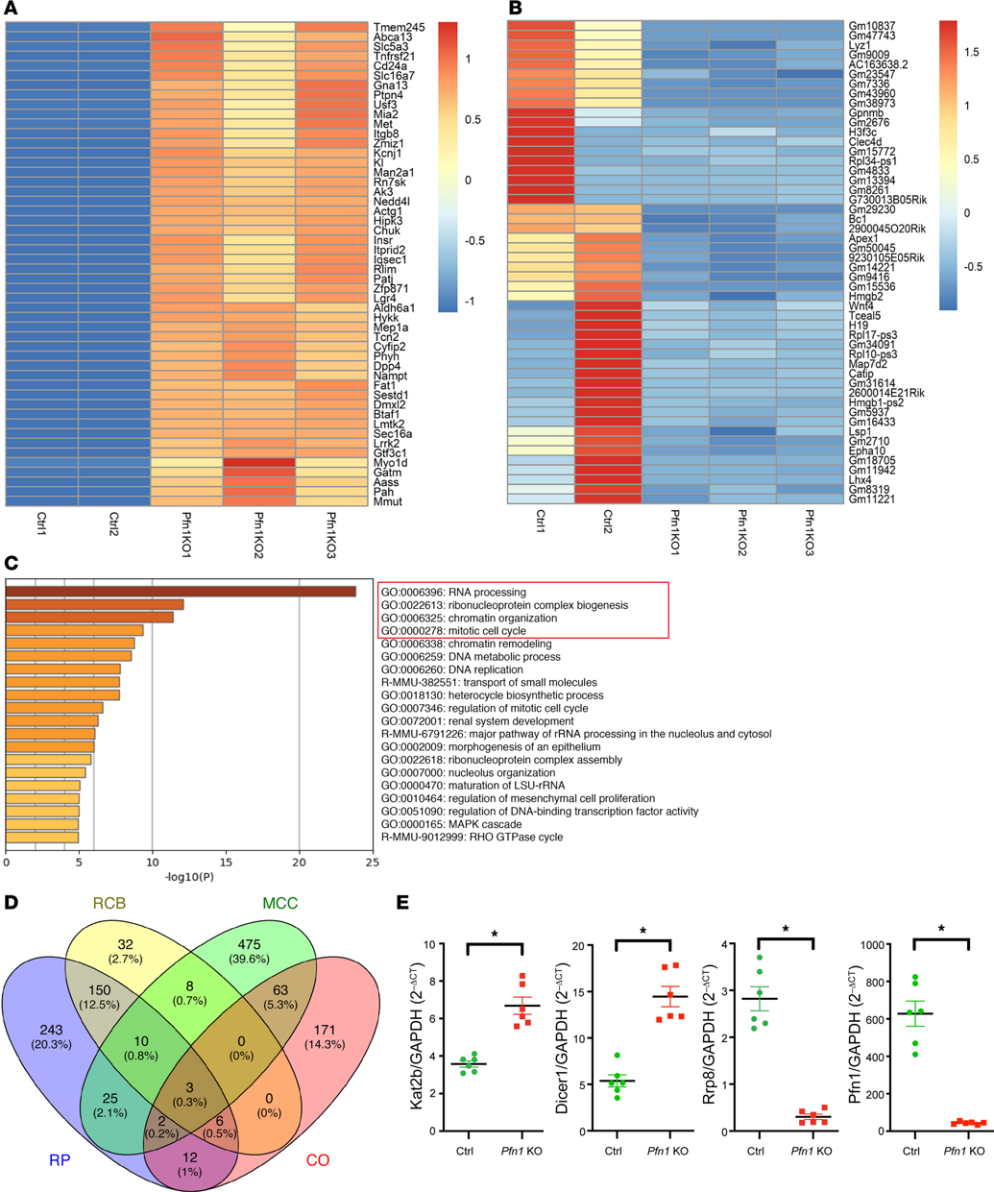
Differentially expressed genes analyzed by podocyte-specific RNA-Seq in *Pfn1*-KO TRAP mice. (**A** and **B**) Heatmap representing color-coding of the highest upregulated (**A**) and downregulated (**B**) differentially expressed genes (DEGs) in podocytes analyzed by Z ratio in *Pfn1*-KO translating ribosome affinity purification (*Pfn1*-KO TRAP) mice compared with control TRAP mice at 3 weeks of age. *n* = 2 samples in control and *n* = 3 samples in *Pfn1* KO. Purified 3-mouse podocyte mixtures were used as 1 sample to ensure enough podocyte yields for RNA-Seq analysis. (**C**) The top significant enriched signaling pathways according to GO and KEGG pathways. The signaling pathways in the red tangle depict the top 4 signaling pathways. (**D**) Three shared DEGs involved in these top 4 signaling pathways were analyzed by Venny 2.1. RP, RNA processing; RCB, ribonucleoprotein complex biogenesis; CO, chromatin organization; MCC, mitotic cell cycle. (**E**) Real-time PCR of the primary control and *Pfn1*-KO podocytes for validation of these 3 candidates DEGs, including *Kat2b*, *Dicer1*, and *Rrp8* from **D**. *n* = 6. *P < 0.05 vs. control. Statistics were analyzed via a 2-tailed *t* test.

Given the observation that *Pfn1*-KO podocytes exhibited MC, podocyte loss, glomerulosclerosis, and kidney failure, we next aimed to understand the potential mechanism underlying this observed phenotype. To this end, we generated *Pfn1-*KO TRAP and control TRAP mice by crossing the TRAP mice with our *Pfn1^fl/+^ Pod-Cre Rosa-DTR^fl^* mice. This approach allowed us to identify the mRNAs that are actively being translated in the TRAP podocyte population by leveraging podocin-driven Cre recombinase-dependent activation of the GFP-tagged L10a ribosomal protein (GFP-L10a) (22, 36). Using *Pfn1-*KO TRAP and control TRAP mice at 3 weeks of age, we performed podocyte-specific RNA-Seq profiling. Gene expression data were analyzed using the fastp tool (Supplemental Table 2). The top 50 differentially expressed genes (DEGs) with the highest upregulated and downregulated in podocytes are displayed in heatmaps (Figure 6, A and B, respectively). Through Gene Ontology (GO) and Kyoto Encyclopedia of Genes and Genomes (KEGG) pathway analysis, we found that RNA processing, ribonucleoprotein complex biogenesis, chromatin organization, and mitotic cell cycle were the most significantly enriched pathways (Figure 6C); this is consistent with the findings observed in the *Pfn*1-KO podocytes that demonstrated aneuploidy and reentry of the cell cycle. After comparing the DEGs with those in the database of mouse genome informatics (MGI; https://www.informatics.jax.org/) regarding the aforementioned 4 most significantly enriched pathways, 274 upregulated DEGs and 177 downregulated DEGs involved in the RNA processing, 119 upregulated DEGs and 90 downregulated DEGs involved in the ribonucleoprotein complex biogenesis, 160 upregulated DEGs and 97 downregulated DEGs involved in chromatin organization, and 451 upregulated DEGs and 135 downregulated DEGs involved in the mitotic cell cycle were observed (Supplemental Table 3). Among these, 3 shared DEGs were identified, which were implicated in these 4 most significantly enriched pathways (Figure 6D). We performed real-time PCR on these 3 shared genes and identified a marked reduction in *Rrp8* (Figure 6E). As such, *Rrp8* reduction could potentially contribute to the observed phenotype of podocytes after the loss of *Pfn1*.

### Rrp8 expression is critical in rescuing Pfn1-KO podocytes.

**Figure 7:**
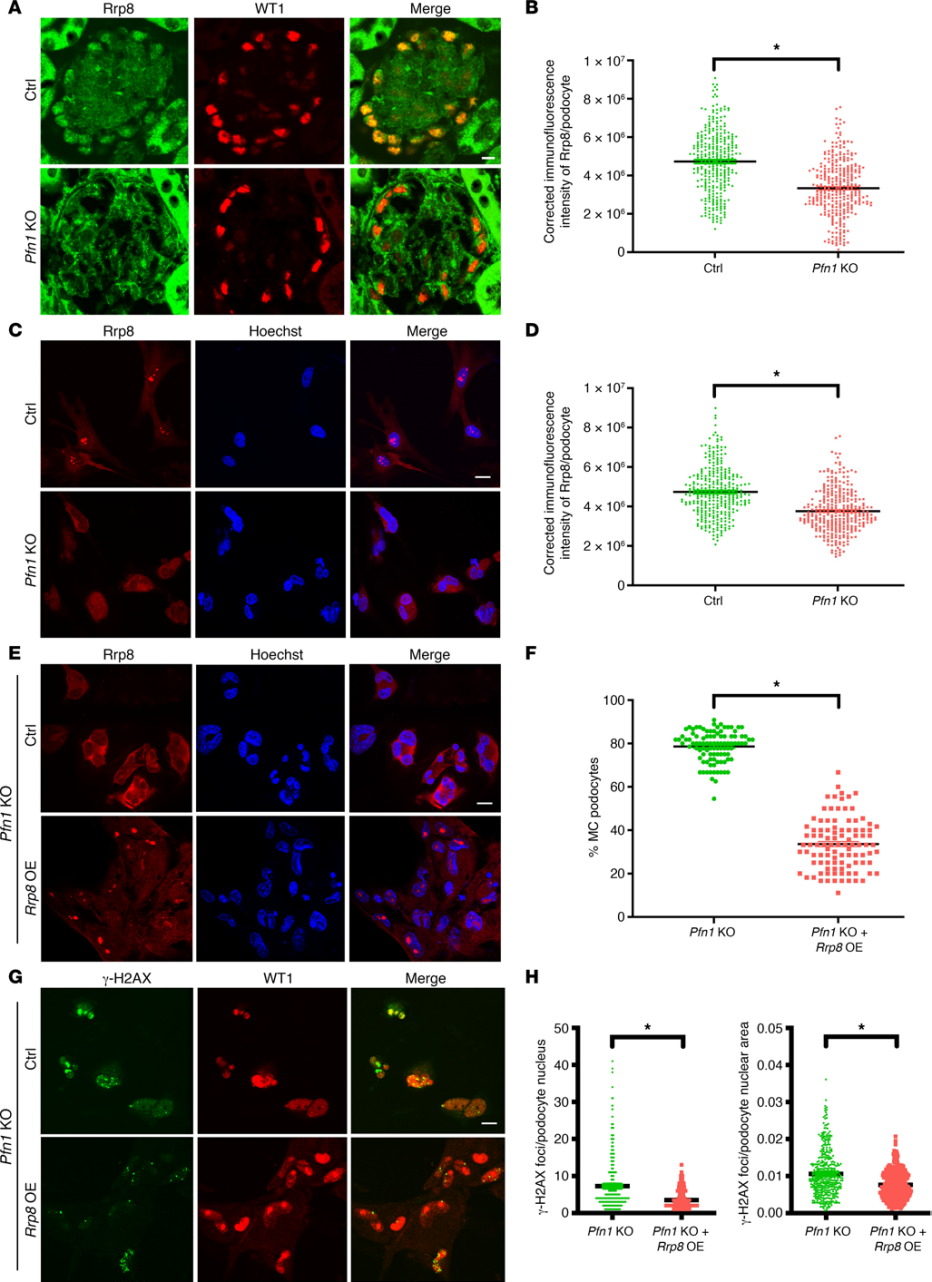
Overexpression of *Rrp8* mitigates DNA damage and improves MC appearance in *Pfn1*-KO podocytes. (**A**) Representative immunofluorescence images of podocytes in control and *Pfn1-*KO glomeruli at 5 weeks of age stained with Rrp8 (green) and WT1 (red). Scale bar: 20 μm. (**B**) Quantification of immunofluorescence intensity of Rrp8 per podocyte in **A**. Total of 320 podocytes in 6 different mice. (**C**) Representative immunofluorescence images of podocytes isolated from control and *Pfn1-*KO mice at P7 stained with Rrp8 (red) and Hoechst (blue). Scale bar: 20 μm. (**D**) Quantification of immunofluorescence intensity of Rrp8 per podocyte in **C**. Total of 320 podocytes in 5 independent experiments. (**E** and **G**) Representative immunofluorescence images of podocyte isolated from *Pfn1-*KO mouse at P7 transduced with mouse *Rrp8* lentiviral activation particles or control particles (Ctrl), followed by staining with Rrp8 (red) and Hoechst (blue), as shown in **E**, or γH2AX (green) and WT1 (red), as shown in **G**. Scale bar: 20 μm. (**F**) Quantification of the percentage of MC podocytes per field of view in **E**. Total of 100 fields of view in 5 independent experiments. (**H**) Quantification of γH2AX foci per podocyte nucleus (left) and per podocyte nuclear area (right) in **G**. Total of 400 cells in 5 independent experiments. *P < 0.05 vs. control. Statistics were analyzed via a 2-tailed *t* test. OE, overexpression.

To further confirm the observed *Rrp8* reduction in our TRAP-RNA-Seq in the *Pfn1*-KO podocytes, we next performed immunofluorescence for Rrp8 protein expression, revealing diminished immunoreactivity of Rrp8 in the glomeruli of *Pfn1*-KO mice when compared with controls (Figure 7A, quantified in Figure 7B). Consistent with this, we also observed a reduction in podocyte Rrp8 expression in the *Pfn1*-KO mouse primary podocytes when compared with controls (Figure 7C, quantified in Figure 7D). To determine whether the reduced expression of Rrp8 plays a critical role in the phenotype observed in *Pfn1*-KO podocytes, we confirmed the overexpression of lentiviral *Rrp8* in *Pfn1*-KO podocytes by immunoblotting (Supplemental Figure 8A). Overexpression of *Rrp8* in *Pfn1*-KO podocytes resulted in a reduction in the number of MC podocytes, (Figure 7E, quantified in Figure 7F), while also rescuing dsDNA damage in the mutant podocytes (Figure 7G, quantified in Figure 7H). To better understand the specific contributions of *Rrp8* overexpression and knockdown in podocyte function, we performed experiments to investigate the effects of *Rrp8* overexpression or knockdown alone in mouse WT control podocytes. Notably, *Rrp8* overexpression or knockdown alone did not show any obvious effects on the occurrence of MC (Supplemental Figure 9A, quantified in Supplemental Figure 9C for *Rrp8* overexpression; Supplemental Figure 9B, quantified in Supplemental Figure 9D for *Rrp8* knockdown). Moreover, neither *Rrp8* overexpression nor knockdown affected the cell cycle phases compared with that in WT control podocytes (Supplemental Figure 7B for *Rrp8* overexpression and Supplemental Figure 7C for *Rrp8* knockdown). However, *Rrp8* knockdown alone, but not *Rrp8* overexpression, resulted in increased DNA damage, as identified by γ-H2AX staining, in comparison to WT control podocytes (Supplemental Figure 9E, quantified in Supplemental Figure 9G for *Rrp8* overexpression; Supplemental Figure 9F, quantified in Supplemental Figure 9H for *Rrp8* knockdown). Collectively, these findings suggest that reduced Rrp8 expression in *Pfn1*-KO mice potentially contributes to the observed phenotypes, including DNA damage and MC. Furthermore, the restoration of Rrp8 expression appears to mediate positive effects in counteracting the consequences of *Pfn1* deficiency in podocytes.

### MC podocytes, reduced expression of profilin1, and podocyturia were present in patients with proteinuric kidney diseases.

**Figure 8:**
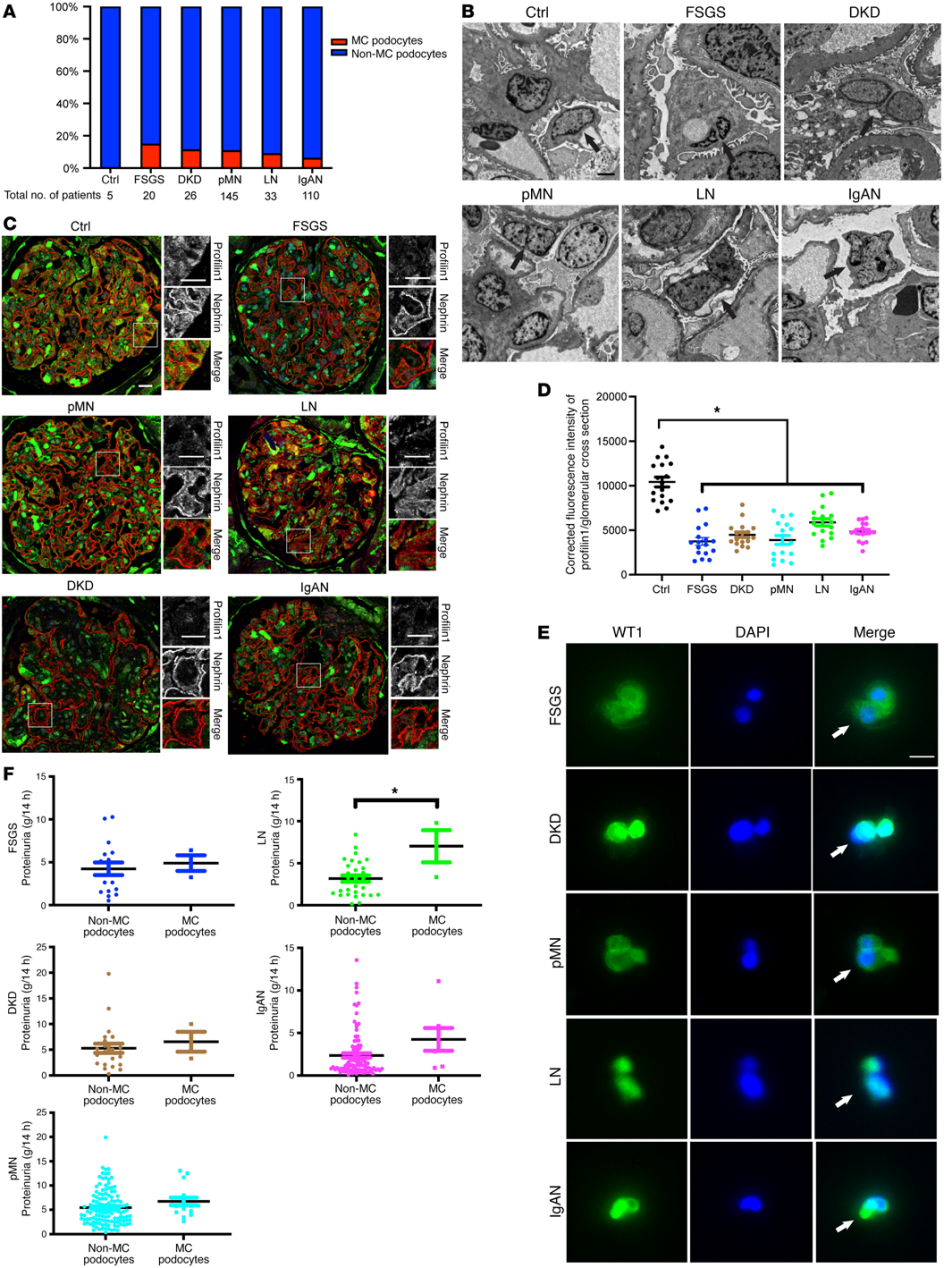
Patients with proteinuric kidney disease demonstrate reduced podocyte PFN1 expression and MC. (**A**) The percentage of patients with MC podocytes observed in kidney biopsy specimens using transmission electron microscopy (TEM) in patients with nonglomerular disease following nephrectomy (control; 0 of 5 patients, 0%), patients with focal segmental glomerulosclerosis (FSGS; 3 of 20 patients, 15%), patients with diabetic kidney disease (DKD; 3 of 26 patients, 11.5%), patients with primary membranous nephropathy (pMN; 16 of 145 patients, 11%), patients with lupus nephritis (LN; 3 of 33 patients, 9.1%), and patients with IgA nephropathy (IgAN; 7 of 110 patients, 6.4%) hospitalized between September 2019 and August 2022. (**B**) Representative TEM images of kidney biopsy specimens. Mononucleated podocytes in control and MC podocytes in patients with FSGS, DKD, pMN, LN, and IgAN are depicted with arrows. Scale bar: 2 μm. (**C**) Representative immunofluorescence images of glomeruli in control patients and patients with FSGS, DKD, pMN, LN, and IgAN with MC podocytes observed with TEM stained with profilin1 (green) and nephrin (red). Scale bar: 20 μm. (**D**) Quantification of C examining mean immunofluorescence intensity of profilin1 in the glomeruli. Total of 17 glomeruli in 3 patients from each group. *P < 0.05. Statistics were analyzed via a 1-way ANOVA with Dunnett’s correction. (**E**) Representative immunofluorescence images of urine samples in patients with FSGS, DKD, pMN, LN, and IgAN with MC podocytes stained with WT1 (green) and DAPI (blue). Arrows denote multinuclei. Scale bar: 20 μm. (**F**) Quantification of urine protein excretion in patients with FSGS, DKD, pMN, LN, and IgAN at the time of kidney biopsy. MC podocytes and non-MC podocytes were observed with TEM individually in each group.*P < 0.05. Statistics were analyzed via a 2-tailed *t* test.

To support the hypothesis that profilin1 disruption may contribute to proteinuric kidney disease in humans, we conducted a retrospective study on patients with biopsy-proven proteinuric kidney diseases, including FSGS, DKD, pMN, LN, and IgAN. Ultrastructural analysis of kidney biopsies demonstrated a presence of MC podocytes in 15% of patients with FSGS (3 of 20), 11.5% of patients with DKD (3 of 26), 11% of patients with pMN (16 of 145), 9.1% of patients with LN (3 of 33), and 6.4% of patients with IgAN (7 of 110) (Figure 8, A and B). To explore the potential contribution between MC podocytes and profilin1 expression, we next performed immunofluorescence staining for profilin1 along with nephrin, a podocyte marker, using the kidney tissues of patients with the observed MC podocyte phenotype. Compared with the control patients without proteinuria who underwent nephrectomy, there was a decrease in profilin1 expression (Figure 8C, quantified in Figure 8D). MC podocytes in the urine of these patients were also observed (Figure 8E). Interestingly, further analysis of clinical characteristics (age, systolic and diastolic blood pressure, serum levels of total protein, albumin, total cholesterol, triglyceride, and serum creatinine at the time of kidney biopsy) of patients with observed MC podocytes compared with those with non-MC podocytes on TEM, revealed no observable difference, except in the patients with LN in which serum total protein and albumin were lower in the patients with MC podocytes (Supplemental Table 4). Proteinuria levels in the patients observed to have MC podocytes demonstrated no overt differences when compared with the patients observed to only have mononucleated podocytes in FSGS, DKD, pMN, and IgAN. However, worsened proteinuria was observed in patients with LN with MC podocytes when compared with those with mononucleated podocytes (Figure 8F).

## Discussion

In this investigation, we uncovered an essential role for profilin1 in maintaining podocyte health. The absence of this gene results in severe proteinuria and foot process effacement, as observed in our genetic mouse model. Although profilin1 has been previously implicated as a key regulator of the actin cytoskeleton (37, 38), our electron tomography revealed an extensive network of MC podocytes lacking *Pfn1*.

As mature podocytes are considered terminally differentiated and therefore unable to divide and replicate, a variety of mechanisms have been proposed to explain its loss after injury (6). To account for the MC podocyte phenotype observed in our *Pfn1*-KO model, we posit that this phenomenon is a result of podocyte dysfunction due to abnormal cell division and subsequent MC, a process of incomplete cell division leading to abnormal chromosomal segregation (39). The MC was first coined in the 1930s when cells with aneuploidy were observed in malignant tissues (7). This phenomenon was later attributed to defective mitosis with respect to chromosomal segregation (40) and was later noted to be a mechanism of cell death in HeLa cells (41).

MC has been also implicated as a modality of podocyte loss, as it has been observed in urinary podocytes from patients with diabetic nephropathy, revealing prominent phosphorylated vimentin, a cellular mitosis marker (8, 42). Furthermore, previous studies have demonstrated that MC occurs following adriamycin-induced glomerular injury in mouse models, which are governed by mouse double minute 2 (MDM2), an E3 ligase that regulates the cell cycle (10). Moreover, podocyte-specific loss of *MDM2* resulted in proteinuria and glomerulosclerosis through the induction of p53 (11). In addition, it has been shown that reduced podocyte-specific myeloid-derived growth factor expression resulted in MC (43).

In our study, to further gain mechanistic insight into how the in vivo and in vitro loss of *Pfn1* results in MC, we were able to take advantage of FUCC2aR technology to visualize podocyte cell cycle reentry. Recently, we reported that podocytes reenter the cell cycle following dsDNA damage and progress to the G_1_ phase, resulting in senescence (12). Interestingly, podocytes devoid of *Pfn1* demonstrate entrance into G_1_, S, and G_2_ phases, along with increased expression of cyclin D1 and B1, which have been shown to be involved in the development of MC (39). These findings suggest that injured podocytes undergo different forms of cell death. Previous findings have shown that chondrocytes lacking *Pfn1* have incomplete abscission during late cytokinesis, causing incomplete daughter cell separation (17). However, when perturbing podocyte profilin1 expression, the mitotic function is likely primarily mediated by this gene, as we did not observe overt defects in stress fiber formation by phalloidin staining. As podocyte effacement is apparent in TEM and SEM images of the mutant mice, a major constraint of our phalloidin experiments is that the primary cells utilized have undergone culturing potentially deviating from in vivo observation. Moreover, in cultivated embryonic chicken fibroblasts, profilin1 exhibited less association with actin filament arcs compared with profilin-2a (PFN2a) when costained with phalloidin (44). Yet, this does not rule out other direct effects of profilin1 on the actin cytoskeleton, as it has been shown to interact with formins, Ena/Vasp, and WAVE (45). However, cell type–dependent differences in function likely exist, as profilin1-deficient keratinocytes demonstrate the failure to repair dsDNA breaks (46), which similarly may induce our mutant cells to continue through the cell cycle, as they are unable to return to the quiescent state.

Examining human biopsy specimens from patients with FSGS, DKD, pMN, LN, and IgAN also demonstrated that MC likely occurs in various forms of glomerular kidney disease. The correlation between podocyte aneuploidy and disease severity of proteinuric kidney disease remains an open question. Our current study is limited by the percentage of patients observed to have MC podocytes, as it ranged between about 5% and 15%. We hypothesize that this could be partly due to sample bias, where there is a limited number of glomeruli observed on a single kidney biopsy specimen sent for electron microscopy. As there was reduced podocyte profilin1 expression in patients with the aforementioned glomerular kidney diseases, we hypothesize that there is likely a higher number of patients who have evidence of podocyte MC than this study was able to capture (Figure 8A). Another limitation of our study is the difficulty of definitively identifying MC cells in kidney biopsy specimens from patients in vivo using immunofluorescence with podocyte markers such as WT1. Under the confocal microscope, it proved challenging to distinguish whether a few WT1-positive nuclei in glomeruli were derived from the same podocyte or different podocytes or if they originated from different layers within the glomerulus. This complexity made it difficult to conclusively identify MC cells in vivo.

Finally, we utilized TRAP technology to perform an unbiased RNA-Seq screen on translating ribosomes specifically in the podocytes to identify a podocyte transcriptomic signature when *Pfn1* is deleted. Pathway analysis of gene expression revealed a striking change in RNA processing, ribonucleoprotein complex biogenesis, chromatin organization, and mitotic cell cycle pathways, confirming the phenotypic changes observed in the *Pfn1*-KO podocytes. Our screen allowed us to identify that *Pfn1*-KO podocytes were partially rescued by overexpression of *Rrp8*, which was unexpected. Given that these are in vitro experiments, it is unclear whether the increase in Rrp8 protein expression prevents approximately 20% of the mononuclear *Pfn1*-KO podocytes from undergoing aneuploidy as in vitro podocytes proliferate. These findings will galvanize future studies to identify whether increased *Rrp8* has a salutary effect in vivo in maintaining podocyte function by preventing MC. The relationship between profilin1 and Rrp8 in the context of MC in podocytes warrants further investigation to gain a deeper understanding of the underlying mechanisms.

In conclusion, our current work suggests that reduced profilin1 expression in murine and human podocytes predisposes podocytes to undergo MC, highlighting its distinct physiological significance in maintaining a normal glomerular filtration barrier.

## Methods

### Antibodies, reagents, and plasmids.

Rabbit anti-profilin1 monoclonal antibody (used for immunofluorescence, Thermo Fisher Scientific, catalog MA5-32683); rabbit anti-profilin1 polyclonal antibody (used for Western blotting, Cell Signaling Technology, catalog 3237S); guinea pig anti-nephrin polyclonal antibody (Progen, catalog GP-N2); rabbit anti-Wilms tumor 1 (WT1) monoclonal antibody (Abcam, catalog ab89901); mouse anti-WT1 monoclonal antibody (Novus Biologicals, catalog NB11-60011); rabbit anti-p21 monoclonal antibody (Abcam, catalog ab188224); mouse anti-p53 monoclonal antibody (Cell Signaling Technology, catalog 2524S); rabbit anti-cyclin monoclonal D1 antibody (Cell Signaling Technology, catalog 2978S); mouse anti-cyclin polyclonal B1 antibody (Cell Signaling Technology, catalog 4138S); mouse anti–Ser 319–phosphorylated γH2AX monoclonal antibody (EMD Millipore, catalog 05-636); rat anti-mCherry monoclonal antibody (Invitrogen, catalog M11217); goat anti-mVenus polyclonal antibody (MyBioSource, catalog MBS448126); Hoechst (Thermo Fisher Scientific, catalog 62249); rabbit anti-GAPDH monoclonal antibody (Cell Signaling Technology, catalog 2118S); mouse anti-GFP monoclonal antibody (Roche, catalog 11814460001); Alexa Fluor 488–conjugated phalloidin (Invitrogen, catalog A12379); Alexa Fluor 594–conjugated phalloidin (Invitrogen, catalog A12381); Alexa Fluor 488–conjugated tubulin (Abcam, catalog ab195883); Alexa Fluor 488 goat anti-mouse IgG antibody (Invitrogen, catalog A11029); Alexa Fluor 488 goat anti-rabbit IgG antibody (Invitrogen, catalog A11034); Alexa Fluor 488 donkey anti-goat IgG antibody (Invitrogen, catalog A11055); Alexa Fluor 594 goat anti-rabbit IgG antibody (Invitrogen, catalog A21207); Alexa Fluor 594 goat anti-mouse (Invitrogen, catalog A11032); Alexa Fluor 594 goat anti-guinea pig (Invitrogen, catalog A11076); Alexa Fluor 594 goat rat (Invitrogen, catalog A11007); Alexa Fluor 647 goat anti-rabbit (Invitrogen, catalog A21245); mouse anti–mouse IgG HRP-conjugated antibody (Rockland, catalog 18-8817-31); and rabbit anti–mouse IgG HRP-conjugated antibody (Millipore Sigma, catalog AP160P) were purchased commercially. Collagen type I (bovine) was purchased from Corning (catalog 354231) for mouse primary podocyte cell culture. Collagen type I (rat tail) (catalog A10483-01) was purchased from Gibco for human podocytes cell line cell culture. Insulin-Transferrin-Selenium (ITS; catalog 41400-045) was purchased from Gibco. Diphtheria toxin (catalog D0564) was obtained from Millipore Sigma. Mouse *Rrp8* lentiviral activation particles (catalog sc-430400-LAC) and mouse *Rrp8* shRNA lentiviral particles (catalog sc-108234-V) were purchased from Santa Cruz Biotechnology. FuGENE transfection reagent (catalog E5911) was purchased from Promega. SlowFade gold antifade mountant with DAPI (catalog S36938) was purchased from Thermo Fisher Scientific. The cell cycle phase determination kit (item 10009349) was purchased from Cayman Chemical. Adriamycin (catalog D1515) was purchased from Millipore Sigma. H_2_O_2_ (item 2186-01) was purchased from Avantor Performance Materials.

### Generation of mice.

*Pfn1^fl/fl^* mice, in which essential coding exon1 is flanked by *loxP* sites, were a gift from Reinhard Fassler (Max Planck Institute, Martinsried, Germany) (17). *Pfn1^fl/fl^* mice were mated with *Pod-Cre Rosa-Dtr^fl^* mice on a C57BL/6 background, a gift from Lloyd Cantley (Yale School of Medicine) (47), to generate *Pfn1^fl/fl^* *Pod-Cre Rosa-Dtr^fl^* (*Pfn1*-KO) mice. *Pfn1^+/+^ Pod-Cre Rosa-Dtr^fl^* mice were used as controls. R26Fucci2aR mice on a BALB/c background were a gift from Ian James Jackson (MRC Human Genetics Unit, University of Edinburgh, Edinburgh, United Kingdom) (12). TRAP mice on a C57BL/6 background were obtained from The Jackson Laboratory (stock 022367). All generated mice were backcrossed more than 5 generations before experiments. Mouse tail genotyping was performed by PCR using gene-specific primers (Supplemental Table 1) and previously described protocols (25, 35, 48, 49).

### Cell culture.

Primary podocytes were isolated from P7 control mice and *Pfn1*-KO or *Pfn1^fl/fl^ Pod-Cre-Rosa-Dtr^fl^ R26Fucci2aR^homozygous/homozygous^* mice (*Pfn1*-KO-*FUCCI* pups) and harvested as described previously in our laboratory (12, 50). Two days after the glomerular cells were seeded on collagen type I–coated (bovine) cell culture dishes, RPMI 1640 medium with L-glutamine, supplemented with 10% FBS, 100 U/mL penicillin/100 μg/mL streptomycin, 100 mM HEPES, 1 mM sodium pyruvate, 1 mM sodium bicarbonate, and 0.1 μg/mL diphtheria toxin was added, and the medium changed every other day until only the primary podocytes remain. The purity of cultured primary podocytes determined by WT1 (a podocyte marker) immunostaining was greater than 98% (data not shown). Primary podocytes at passage 1 or 2 were used in experiments.

The conditionally immortalized human *PFN1-*KO podocyte cell line was generated using CRISPR/Cas9 (Applied Biological Materials) transfected with FuGENE as previously described (51). Clones were obtained by limited dilution and expanded under puromycin selection. The human *PFN1*-KO podocyte cell line was confirmed by Western blotting and real-time PCR analysis. Cells were cultured as previously described (52). In brief, immortalized human podocytes grown permissively at 33°C in RPMI 1640 medium supplemented with L-glutamine, 10% FBS, ITS supplement, 100 U/mL penicillin, 100 μg/mL streptomycin for cellular proliferation were thermoshifted to 37°C in cell culture medium without ITS for approximately 10–14 days for cellular differentiation. All tissue culture dishes were coated with collagen type I (rat tail).

### Kidney histology and quantification.

Mice were anesthetized by intraperitoneal injection of ketamine and xylazine, followed by circulatory perfusion with PBS and subsequent fixation with 4% paraformaldehyde (PFA) through the left ventricle, either with or without 2% glutaraldehyde, for kidney histology and TEM or immunofluorescence, respectively. The 4 μm kidney sections were used for histopathology analysis with a light microscope and immunofluorescence staining, respectively. For histology, kidney sections were sent to the Yale Pathology Core Tissue Service for&E, periodic acid–Schiff, and Masson’s trichrome staining. To assess glomerulosclerosis, glomerular mesangial expansion, and the severity of renal tubulointerstitial lesions, kidney sections were assessed as previously described (13, 50). Briefly, the severity of glomerulosclerosis or glomerular mesangial expansion in each glomerulus was scored on periodic acid–Schiff–stained sections in a double-blinded manner as follows: 0, no sclerosis or mesangial expansion; 1, sclerosis or mesangial expansion of <10% of the glomeruli; 2, sclerosis or mesangial expansion of 10%–25% of the glomeruli; 3, sclerosis or mesangial expansion of 25%–50% of the glomeruli; and 4, sclerosis or mesangial expansion of >50% of the glomeruli. The renal tubulointerstitial lesions, defined as tubular dilation, tubular atrophy, proteinaceous cast formation, and interstitial fibrosis, were assessed on trichrome-stained sections using Masson’s trichrome–stained kidney sections as follows: 0, no lesion; 1, lesion of <10% of the area; 2, lesion of 10%–25% of the area; 3, lesion of 25%–50% of the area; and 4, lesion of >50% of the area. SEM was performed by the CCMI Electron Microscopy Core Facility at Yale School of Medicine. Briefly, the mouse kidney tissue samples were fixed in a solution containing 2.5% glutaraldehyde and 2% PFA in 0.1 M sodium cacodylate buffer at pH 7.4 overnight. After rinsing, the samples were post-fixed with 1% osmium tetroxide in 0.1 M cacodylate buffer for 1 hour. Following another round of rinsing in distilled water, the samples were dehydrated through an ethanol series, starting at 30%, then progressing through 50%, 70%, 95%, and, finally, 100% ethanol. Subsequently, the dehydrated samples were transferred into a Leica 300 critical point dryer, where liquid carbon dioxide was used as the transitional fluid for the drying process, which took approximately 3 hours. The samples were then carefully oriented and attached to aluminum stubs using carbon graphite. To enhance conductivity, the samples were sputter coated with a 5 nm layer of platinum 80 and palladium 20, using a Cressington 208HR sputter coater. Finally, digital images of the samples were acquired using a Zeiss CrossBeam 550 scanning electron microscope, operating at an accelerating voltage between 1.5 and 2 kilovolts (kV). For quantitative analysis of ultrastructural changes of glomeruli examined by TEM, the number of foot processes in each glomerular capillary and the thickness of the GBM were quantified using NIH ImageJ software, as previously described (50).

### Immunofluorescence.

Freshly isolated mouse primary podocytes or differentiated immortalized human podocytes were seeded on collagen type I–coated coverslips, washed with 1X PBS buffer, and fixed with 4% PFA for 20 minutes at room temperature, followed by permeabilization with 0.1% Triton X-100 for 20 minutes at room temperature. Human kidney biopsy paraffin-embedded sections were first deparaffinized with xylene, followed by hydration with a decreasing gradient concentration of ethanol, as previously described (50). Mouse kidney cryosections or deparaffinized human kidney sections (4 μm) were subjected to antigen retrieval using 10 mM sodium citrate buffer with 0.05% Tween 20 (pH 6.0) for 10 minutes. After antigen retrieval, slides, and coverslips were blocked with 3% BSA in 1X PBS for 1 hour at room temperature and then incubated with appropriate primary antibodies using optimized dilution at 4°C overnight. After incubation, the slides were washed with 1X PBS 3 times, followed by incubation with the appropriate Alexa Fluor 488–, 594–, and/or 647–conjugated secondary antibodies and/or Hoechst-conjugated (cell-permeable DNA stain marker) antibody as appropriate and then mounted with DAPI Slowfade. Images were acquired using an Andor CSU-WDi spinning disk confocal microscope equipped with a Nikon Ti-E CFI Plan Apochromat Lambda 60× oil immersion objective for immunofluorescence analysis, and images were processed using NIH ImageJ software or Adobe Photoshop 2022. For quantification of podocyte density, 40 glomeruli from 5 mice in each group were evaluated by counting the WT1-positive nuclei in each glomerulus and then normalizing the WT1-positive count in relation to the calculated 3D volume of the glomerular tuft, as previously described (12, 53). For quantification of profilin1 expression in the human kidney biopsy tissue or podocyte Rrp8 expression in vitro and in vivo, the protein staining signal intensity (I_protein_) was measured for the integrated intensity and area, and background intensity was measured by averaging the 3 different areas closest to the defined glomerulus or podocytes in the same field of view without specific staining (I_BK_). The corrected intensity of each glomerulus or each podocyte was determined by subtracting I_BK_ × area (A) from I_protein_ (50). To objectively quantify the number of γH2AX foci per cell, a custom ImageJ macro was used, automating a series of commands (see attached ImageJ macro file in Supplemental Table 5). In brief, freshly isolated podocytes from WT control mice or *Pfn1*-KO mice, as well as WT podocytes after transduction with mouse *Rrp8* lentiviral activation particles (overexpression) or mouse *Rrp8* shRNA lentiviral particles (knockdown), were utilized. The podocytes were stained with γH2AX and WT1, where WT1 served as the podocyte nuclear marker. Subsequently, each image was split into individual channels. The WT1 channel underwent conversion into a binary image, enabling the determination of each nuclear area. The γH2AX channel was similarly converted, and subsequent particles within each cell were automatically calculated to determine the foci number and nuclei area in each podocyte.

### Biochemical measurement of plasma creatinine, urine albumin, and urine creatinine.

Mouse urine albumin levels were measured in duplicate for each sample using an albumin ELISA quantification kit according to the manufacturer’s protocol (Bethyl Laboratories Inc.) at an absorbance of 450 nm. Urine and plasma creatinine levels were measured in duplicate for each sample at the indicated time points above using a colorimetric quantification kit (Bioassay Systems) at an absorbance of 490 nm (Synergy LX Multi-Mode Reader, BioTek).

### Western blotting.

Mouse primary podocytes, *Pfn1*-KO podocytes transduced with mouse *Rrp8* lentiviral activation particles, or differentiated immortalized human podocytes were lysed in lysis buffer containing 50 mM Tris-hydrochloride (pH 7.6), 500 mM sodium chloride, 0.1% SDS, 0.5% deoxycholate, 1% Triton X-100, 0.5 mM magnesium chloride, phosphatase inhibitor cocktail, and protease inhibitor cocktail (Roche, catalog 11697498001). Protein concentrations were quantified using the Pierce BCA Protein Assay Kit (Thermo Fisher, 23225). Equal amounts of sample protein were denatured for 10 minutes at 95°C, followed by loading to each lane, separation via gel electrophoresis on 4%–20% gradient SDS-PAGE gels, and transfer to a PVDF membrane (Millipore Sigma). The membrane was blocked with 5% nonfat milk (American Bio) or 3% BSA (Millipore Sigma) in Tris-buffered saline and 0.05% Tween-20 and incubated with appropriate primary antibodies at 4°C on a shaker overnight. The appropriate HRP-conjugated secondary antibodies were added, and signals were detected using enhanced chemiluminescence reagents (Bio-Rad) exposed using Odyssey (LI-COR Biosciences). For quantification, densitometry was performed using the NIH ImageJ software. See complete unedited blots in the supplemental material.

### Clinical data.

Patients with biopsy-proven proteinuric kidney diseases, including FSGS, DKD, pMN, LN, or IgAN, hospitalized in the Department of Nephrology, the Second Affiliated Hospital of Xi’an Jiaotong University, between September 2019 and August 2022 were retrospectively assessed. In this study, 20 patients with FSGS, 26 patients with DKD, 145 patients with pMN, 33 patients with LN, and 110 patients with IgAN who had complete clinical data, glomeruli visualized under TEM, and retained urine samples during the hospitalization period were enrolled.

### Determination of MC.

MC is a sequence of events resulting from aberrant mitosis and is defined using the following criteria, as previously reported (6–8, 26). Criteria include the presence of multinucleation, irregular nuclei, micronuclei, abnormal mitotic spindles, or denucleation in podocytes. To quantify podocytes with MC in patients, glomerular TEM images from kidney biopsy samples of individuals with various proteinuric kidney diseases were meticulously examined by an experienced renal pathologist. If MC was observed in the podocytes within the glomerulus, the patient’s sample was categorized into the “MC podocytes” group; otherwise, it was grouped into the “non-MC podocytes” group. For the quantification of in vitro podocytes, the percentage of podocytes with MC per field of view was calculated. Subsequently, the percentages of podocytes with MC were compared, and statistical analysis was conducted to determine the significance of any observed differences.

### Urine podocytes.

For mice, aliquots of freshly collected urine samples from the *Pfn1-*KO-FUCCI and control mice were sedimented on type I–collagen coated coverslips and air dried for 20 minutes. The urine samples were fixed with 4% PFA for 20 minutes at room temperature; washed with 1X PBS; permeabilized with 0.1% Triton X-100 for 20 minutes at room temperature; blocked with 5% BSA; incubated with rat anti-mCherry monoclonal antibody (1:100 dilution), goat anti-mVenus polyclonal (1:100 dilution), and rabbit anti-WT1 monoclonal antibodies (1:100 dilution) at 4°C overnight in a humidified chamber; washed with 1X PBS; incubated with the appropriate secondary antibodies at room temperature for 1 hour; mounted with antifade mounting medium with DAPI; and analyzed by confocal microscopy.

For patients, 10 mL urine samples were centrifuged at 1,500*g* for 10 minutes. The excess supernatant was discarded, and 0.1 mL sediment was reserved for making a thin smear on the slide. Air drying of sediments on type I–coated coverslip glass slides was performed, and slides were fixed with 95% alcohol for 15 minutes and blocked with 5% nonfat milk. Samples were incubated with the rabbit anti-WT1 monoclonal antibody (1:100 dilution) at 4°C overnight, followed by incubation with the Alexa Fluor 488–conjugated secondary antibody (1:200 dilution) at room temperature for 1 hour, mounted with antifade mounting medium with DAPI and analyzed by confocal microscopy.

### Cell adhesion, spreading, and wound healing migration assay.

The adhesion assay using crystal violet staining was performed as previously described (14). Mouse primary *Pfn1-*KO and control podocytes or differentiated human *PFN1-*KO and control podocytes (incubation at 37°C for 10 days) were counted by using an automated cell counter (Logos Biosystems Inc., model LUNA-FL), and equal amounts of living cells in each well were seeded on a collagen type I–coated 96-well plate. The cells were trypsinized for 0 minutes, 5 minutes, and 10 minutes, respectively. Nonadherent cells were removed by gentle washing with 1X PBS. Then, the cells were fixed in 95% ethanol for 20 minutes. Fixed cells were stained with 0.1% crystal violet (Millipore Sigma) for 15 minutes at room temperature, washed gently in distilled water 3 times to remove the unstained crystal violet, and lysed in 0.2% Triton X-100 while shaking until a uniform color was obtained. The absorbance of dissolved crystal violet was measured using a microplate reader at 595 nm.

The cell spreading assay was conducted as previously reported in our laboratory (14). Freshly isolated primary podocytes from control and *Pfn1*-KO mice on P7, or human control and *PFN1*-KO podocytes, were seeded in collagen type I–coated 60 × 15 mm tissue culture dishes. Phase-contrast microscopy with a Nikon Eclipse TE200 equipped with Hoffman modulation and Spot RT camera (Diagnostic Instruments) was used to capture images at the beginning of seeding (0 hour), 1 hour after culture, and 2 hours after culture. The cell area at different time points was analyzed using NIH ImageJ software in a masked manner, randomly examining 25 cells per genotype for each experiment. Four independent experiments were performed.

The wound healing migration assay was performed as previously reported protocol (54). Freshly isolated primary podocytes from control and *Pfn1-*KO mice on P7, or human control and *PFN1-*KO podocytes, were seeded in collagen type I–coated 60 × 15 mm tissue culture dishes. A sterile pipette tip was used to create a central wound by scratching the cell monolayer. Subsequently, the cells were allowed to heal for 18 hours before being imaged using phase-contrast microscopy with a Nikon Eclipse TE200 equipped with Hoffman modulation and Spot RT camera. The percentage of wound healing was quantified using NIH ImageJ software. Four independent experiments were performed.

### Pfn1-KO TRAP mice — podocyte mRNA isolation and RNA-Seq analysis.

Purification of podocyte mRNA of *Pfn1-*KO TRAP mice at 3 weeks of age was performed as previously described (22, 49). Briefly, mice were anesthetized and perfused with DEPC-treated 1X PBS through the left ventricle. Kidneys were harvested, and the cortex was carefully dissected, minced into small pieces, and transferred to a prechilled RNase-free kontes pestle with tissue lysis buffer for gentle homogenization (Con-torque, Power-unit, Eberbach). NP-40 and 1,2 -diheptanoyl-sn-glycero-3-phosphocholine (850306P-1g, Avanti Polar Lipids Inc.) were added, followed by centrifugation 2,000*g* and 17,200*g* at 4°C for 10 minutes, respectively. The supernatant from the lysate was prepared by immunoprecipitation (55). The affinity matrix was prepared using Streptavidin Myone T1 dynabeads (catalog 65601, Invitrogen) and biotinylated recombinant protein L (catalog 29997, ThermoFisher Scientific) with GFP antibodies 19C8 and 19F7 (Htz-GFP-19C8 and Htz-GFP-19F7, Antibody and Bioresource Core Facility/Memorial Sloan-Kettering Cancer Center, New York, New York, USA). Freshly resuspended GFP antibodies-affinity matrix in 0.15 M KCl IP wash buffer were added to the post mitochondrial supernatant and gently end-over-end mixed at 4°C overnight. The beads were collected with a magnet, followed by washing using the 0.35 M KCl IP wash buffer to remove the unbound fractions. Then, the beads were resuspended with RLT lysis buffer containing β-mercaptoethanol and recollected using the magnet. The supernatant/bound RNA fraction was extracted and cleaned using the RNeasy kit and RNeasy MiniElute Cleanup Kit (Qiagen) following the manufacturer’s instructions. RNA quantity and quality were determined (Nanodrop Technologies). The appropriate amount of RNA samples with OD 260/280 greater than 1.9 and RNA integrity number (RIN) value greater than 7 were used for RNA-Seq analysis (Yale Center for Genome Analysis). In order to obtain enough podocyte mRNA samples for RNA-Seq analysis, each sample contained the isolated and cleaned podocyte mRNA samples from 3 mice. Two control TRAP mouse samples and 3 *Pfn1*-KO TRAP mouse samples were collected and analyzed.

RNA-Seq was performed in all TRAP samples. The analysis was performed as previously described (12, 22). Briefly, the raw TRAP-Seq fastq files were processed using fastp tool (version 0.20.0). Sequencing reads with low-quality bases were trimmed or filtered using the default setting. Alignment was performed for cleaned reads using STAR (version 2.7.9) and mouse reference genome (gencode version GRCm38.p6 with vM25 gene annotation). Expression quantification was performed for alignment results using featureCounts (version 2.0.0). As genes with low expression levels that represent noise were excluded before downstream analysis, we defined low expression filtering as the presence of ≥6 read counts in at least 20% of samples of expressed genes. The filtered read counts matrix was then normalized using the transcripts per million method. Detection of DEGs in control TRAP and *Pfn1*-KO TRAP mRNA samples was performed using R package DESeq2 (version 1.30.1); the Benjamini-Hochberg procedure was used for multiple test correction; and FDR ≤ 0.05 was used as a threshold for detection of DEGs. The Database for Annotation, Visualization, and Integrated Discovery (DAVID Bioinformatics Resources, https://david.ncifcrf.gov/tools.jsp) was used to identify functional categories for the DEGs enriched in podocytes according to the GO and KEGG pathway analysis. The most significant signaling pathways among the control TRAP or *Pfn1*-KO TRAP mice were produced and graphed using Metascape, a gene annotation and analysis resource (https://metascape.org/gp/index.html#/main/step1). The DEGs in the 4 top signaling pathways were screened and selected by comparison with the MGI database, and the shared DEGs in the top 4 signaling pathways were produced and graphed by using Venny software (https://bioinfogp.cnb.csic.es/tools/venny/index.html).

### Quantitative real-time PCR analysis.

Total RNA was extracted from the primary podocytes from *Pfn1*-KO and control mice (P7) or differentiated human *PFN1*-KO and control podocytes by using the RNeasy kit (Qiagen). The RNA was measured by spectrophotometry (Nanodrop Technologies), and 2 μg total RNA was used for reverse transcription by a high-capacity cDNA Reverse Transcription Kit (Applied Biosystems, catalog 4368814) according to the manufacturer’s instructions. The quantitative PCR amplifications were performed using Power SYBR Green PCR Master Mix (Applied Biosystems) with a 7300 real-time PCR machine. The primers used for real-time PCR are listed in Supplemental Table 1.

### Transduction with lentivirus.

The cultured *Pfn1*-KO primary podocytes were transduced with mouse *Rrp8* lentiviral activation particles or control lentiviral activation particles according to the manufacturer’s instructions (Santa Cruz Biotechnology). The transduction efficiency was approximately 70%–80%, as determined by immunofluorescence (data not shown).

### TUNEL assay.

Apoptotic podocytes in the fresh kidneys of control and *Pfn1*-KO mice at 4 weeks of age were identified using the TUNEL assay (Roche Diagnostics GmbH) according to the manufacturer’s instructions. Podocytes were identified by costaining with WT1 antibody. TUNEL-positive podocytes per glomeruli in 5 mice in each group were counted.

### SA-β-gal activity staining for senescence analysis.

The SA-β-gal activity in freshly isolated primary podocytes or snap-frozen kidney cryosections from control and *Pfn1*-KO mice was examined using an SA-β-gal staining kit (catalog 9860, Cell Signaling Technology), according to the manufacturer’s instructions. Counterstaining of kidney sections was performed with nuclear fast red. The primary podocytes isolated from histone deacetylase 1/2 (*Hdac1/2*) double-KO mice in our laboratory served as a senescence-positive control (12).

### Cellular cell cycle flow cytometric analysis.

The cell cycle determination for in vitro podocytes was assayed using the cell cycle phase determination kit following the manufacturer’s instructions (Cayman Chemical). Briefly, freshly isolated control and *Pfn1*-KO primary mouse podocytes at P7, as well as WT podocytes after transduction with mouse *Rrp8* lentiviral activation particles (overexpression) or *Rrp8* shRNA lentiviral particles (knockdown), were collected using 0.05% trypsin to create a cell suspension, and cell counting was performed using an automated cell counter (Luna Dual fluorescence cell counter, model LUNA-FL). Next, the cells were fixed and permeabilized by placing them in precooled cell cycle phase determination fixative at –20°C overnight. Afterward, the cells were centrifuged at 500*g* at 4°C for 10 minutes and washed twice with cell-based assay buffer. Subsequently, the cells were suspended in 500 μL cell-based assay buffer containing RNase A and propidium iodide provided in the kit. Following a 60-minute incubation in the dark at room temperature, the fluorescence of the propidium iodide–DNA complex was detected using the LSR II flow cytometer (Beckman Coulter Inc.). The cell cycle distribution in each sample was then analyzed via FlowJo software, using the Watson Pragmatic algorithm (Scripps Research) to determine the cell distribution at different stages of the cell cycle.

### Statistics.

All data are presented as mean ± SEM. The number of replicates for each experiment is shown in the figure legends. Statistical analysis was calculated using GraphPad Prism 9.0 software. Comparison between groups was performed using a 2-tailed *t* test or 1-way ANOVA as appropriate and is shown in the figure legends. All *P* values of less than 0.05 were considered statistically significant, as indicated in the text.

### Study approval.

All animal experiments were carried out in accordance with the principles and procedures outlined in the NIH’s *Guide for the Care and Use of Laboratory Animals* (National Academies Press, 2011) and approved by the Institutional Animal Care and Use Committee at Yale University (New Haven, Connecticut, USA). Studies involving human participants were reviewed and approved by the Medical Ethics Committee of the Second Affiliated Hospital of Xi’an Jiaotong University, and the approval number was 2023237. Written informed consent for this study was obtained from all participants.

### Data availability.

All RNA-Seq data were deposited in the GEO database (accession GSE227414). Values for all data points in graphs are reported in the Supporting Data Values file.

## Author contributions

SI, XT, and CEP conceptualized the study. KL, XM, and XT were responsible for the clinical data collection and analysis. RF was responsible for the supervision of the clinical data. JG, XT, and YZ were responsible for RNA-Seq data curation. HZ was responsible for the formal analysis. CEP, AL, and ML were responsible for the construction of the human *PFN1*-KO podocyte cell line using CRISPR/Cas9 technology. SP evaluated the mice’s kidney histology. XT, CEP, WL, PXMR, ES, PB, GL, JP, AP, SN, and MCM were responsible for the investigation. SI was responsible for the funding acquisition. SI and XT wrote the draft of the manuscript. All coauthors reviewed and edited the manuscript.

## Supplementary Material

Supplemental data

Unedited blot and gel images

Supplemental table 2

Supplemental table 3

Supporting data values

## Version 1 10/17/2023

In-Press Preview

## Version 2 12/15/2023

Electronic publication
