# Supplementary material for: Profilin1 is required for prevention of mitotic catastrophe in murine and human glomerular diseases
Source: J Clin Invest. 2023 Dec 15;133(24):e171237. doi: 10.1172/JCI171237 (PMC10721156; doi:10.1172/JCI171237)
Supplement: Supplemental data [file jci-133-171237-s212.pdf]

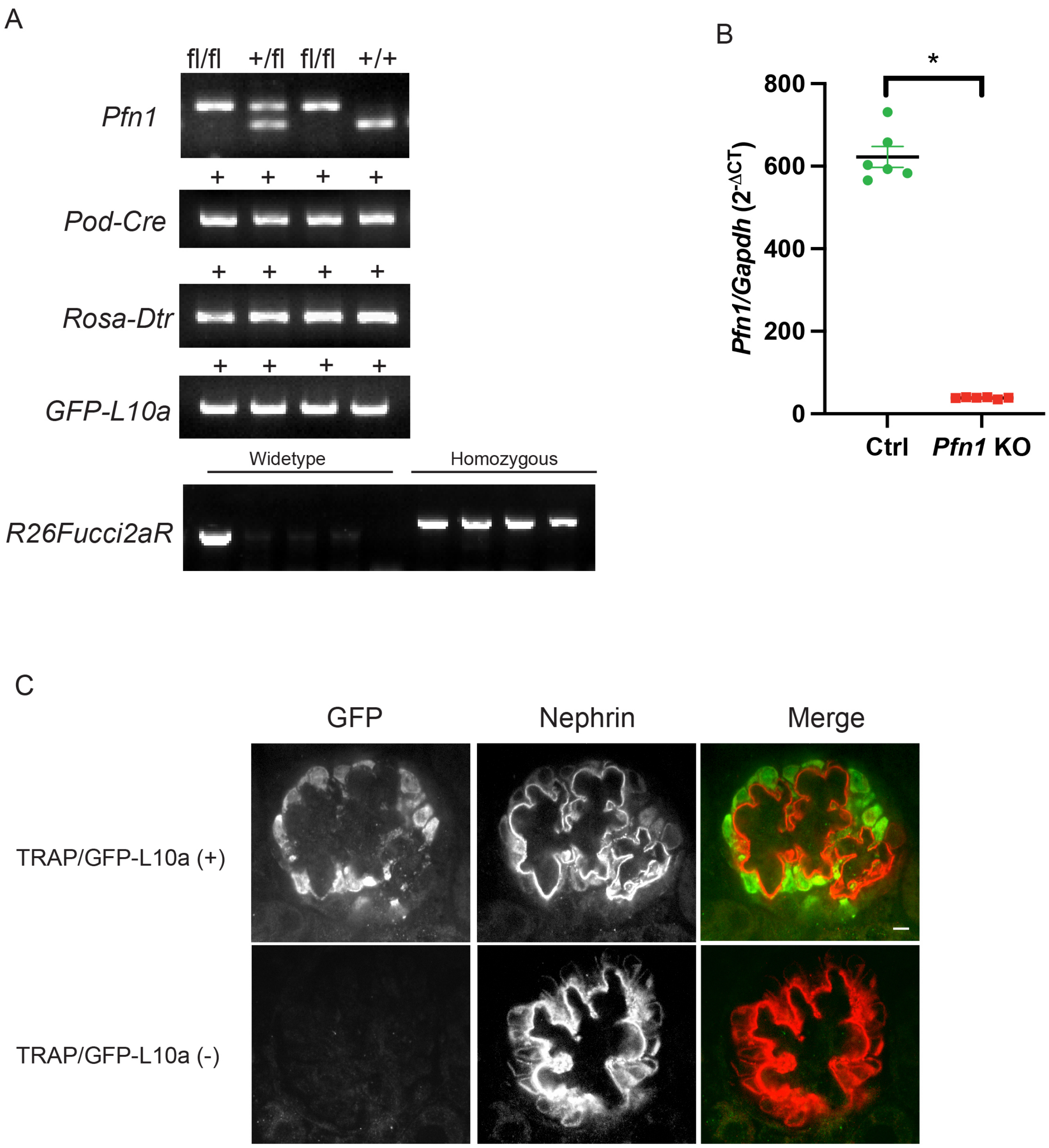

Supplemental Figure 1. Podocyte-specific deletion of *Pfn1* in *Pfn1* KO mice and podocyte-specific expression of GFP(GFP) in TRAP mice. (A) Representative genotyping images of *Pfn1*$^{fl/fl}$, *Pfn1*$^{+/fl}$, *Pfn1*$^{+/+}$, *Podocin-Cre*, *Rosa-Dtr*^{fl}, *GFP-L10a*, *R26Fucci2aR* from the genomic DNA of the mice tail samples at P5. **(B)** Real-time PCR of primary podocytes isolated from the Ctrl and *Pfn1* KO at P7 for validation of deletion of *Pfn1* in podocytes. n= 6 mice. $^*P < 0.05$ vs Ctrl. Statistically analyzed via a 2-tailed *t* test. **(C)** Representative immunofluorescence images of GFP (green) and nephrin (red) on kidney sections of the TRAP (podocyte-specific GFP-L10a) positive mice (upper panel) and TRAP negative mice (lower panel) demonstrate GFP-tagged large ribosome subunit protein L10a in podocytes. Scale bars: 20μm.

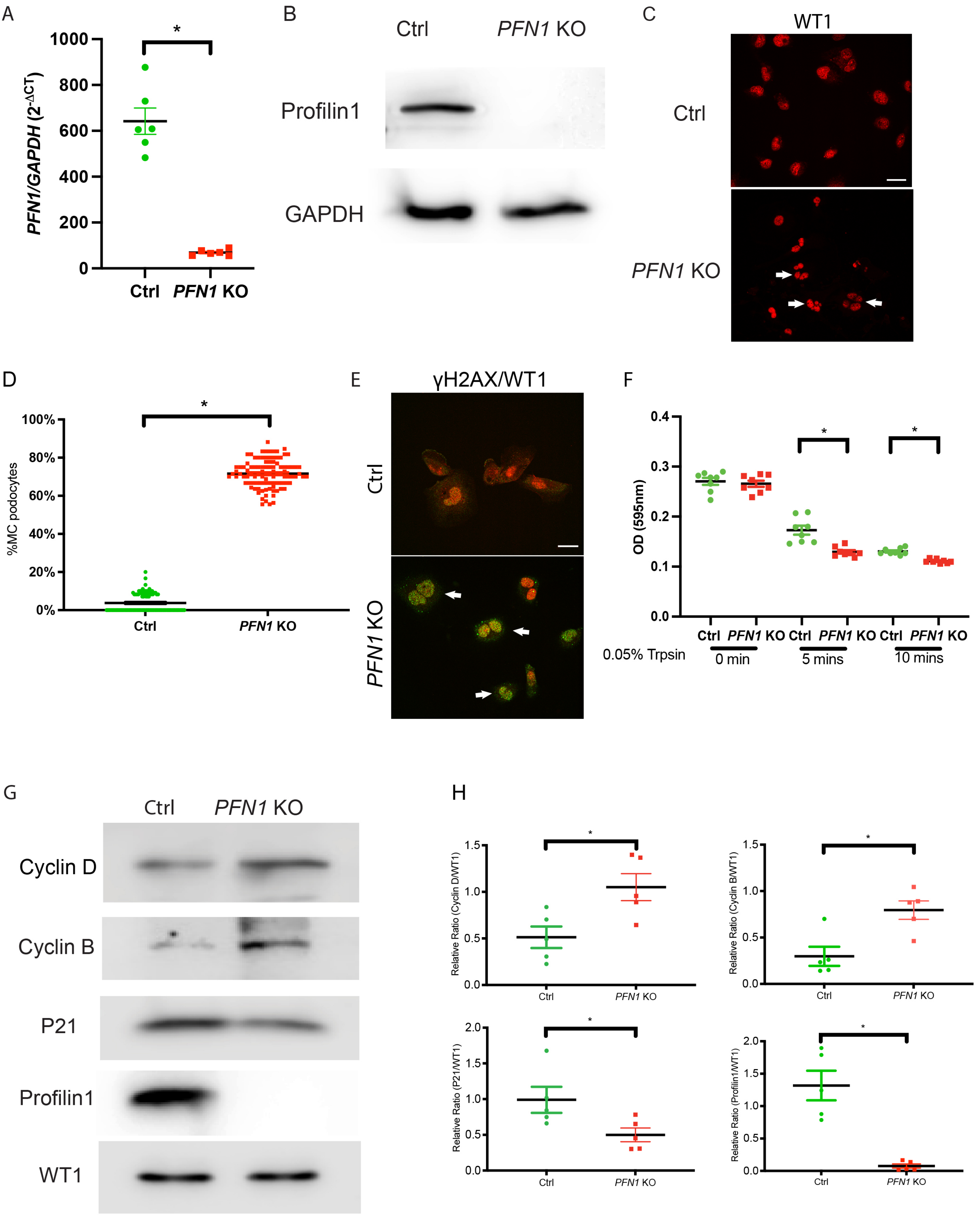

Supplemental Figure 2. Loss of *PFN1* in human podocyte cell line using CRISPR/Cas9 results in MC, dsDNA damage, decreased ability of adhesion, and cell-cycle entry. (A) Identification of *PFN1* deletion by Real-time PCR of the Ctrl and *PFN1* KO podocytes. n= 6 independent experiments. *$P < 0.05$ vs Ctrl. Statistically analyzed via a 2-tailed *t* test. **(B)** Identification of profilin1 loss by Western blotting of the Ctrl and *PFN1* KO podocytes. **(C)** Representative immunofluorescence images of human Ctrl and *PFN1* KO podocytes stained with WT1 (red) showed abnormal MC podocytes in *PFN1* KO podocytes, as indicated by the arrow, compared to Ctrl podocytes. Scale bar: 20μm. **(D)** Quantification of the percentage of MC podocytes per field of view in **C**. Total 100 fields of view in 5 independent experiments. **(E)** Representative immunofluorescence images of human Ctrl and *PFN1* KO podocytes stained with γ-H2AX (green) and WT1 (red), showed increased γ-H2AX expression in *PFN1* KO podocytes, as indicated by the arrow, compared to Ctrl podocytes. Scale bar: 20μm. **(F)** Human Ctrl (green) and *PFN1* KO podocytes (red) after incubation at 37 °C (nonpermissive temperature condition) for 10 days for differentiation demonstrated a significant decrease in adhesion as measured by optic density (O.D). n=8 independent experiments. **(G)** Representative immunoblot images of Cyclin D1, Cyclin B1, P21, profilin1, and WT1 as loading control in Ctrl and PFN1 KO podocytes. **(H)** Quantification of immunoblots in **G**. n=5 independent experiments. *$P < 0.05$ vs Ctrl. Statistically analyzed via a 2-tailed *t* test.

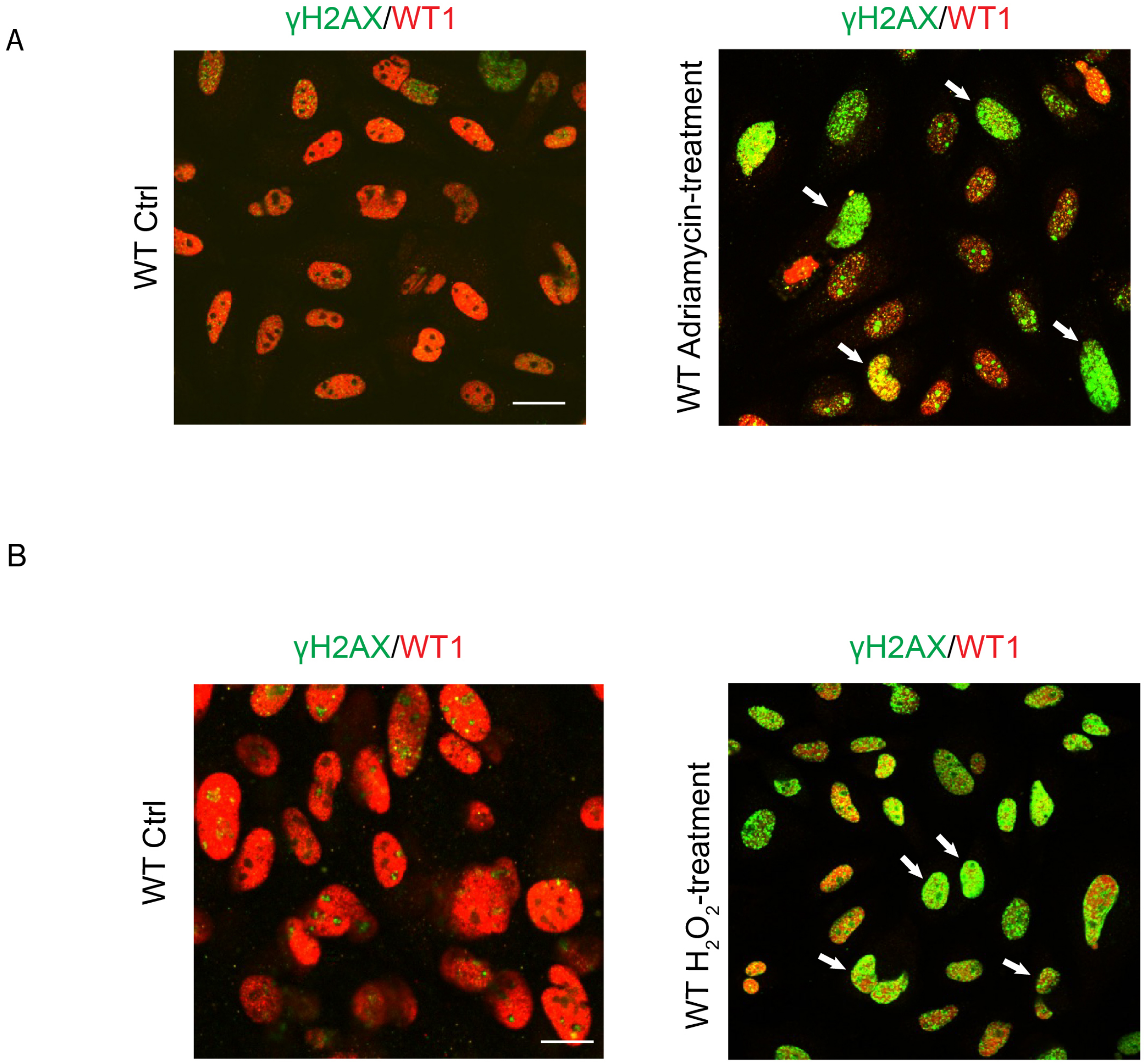

Supplemental Figure 3. Positive control of γH2AX expression in podocytes. (A) Representative immunofluorescence images of Ctrl and Adriamycin-treated mouse wild type (WT) podocytes stained with γH2AX (green) and WT1 (red) showed higher expression levels in Adriamycin-treated podocyte nuclei, as indicated by the arrow, compared to Ctrl podocytes. **(B)** Representative immunofluorescence images of Ctrl and H_2O_2-treated mouse WT podocytes stained with γH2AX (green) and WT1 (red) showed higher expression levels in H_2O_2-treated podocyte nuclei, as indicated by the arrow, compared to Ctrl podocytes. Scale bar: 20μm.

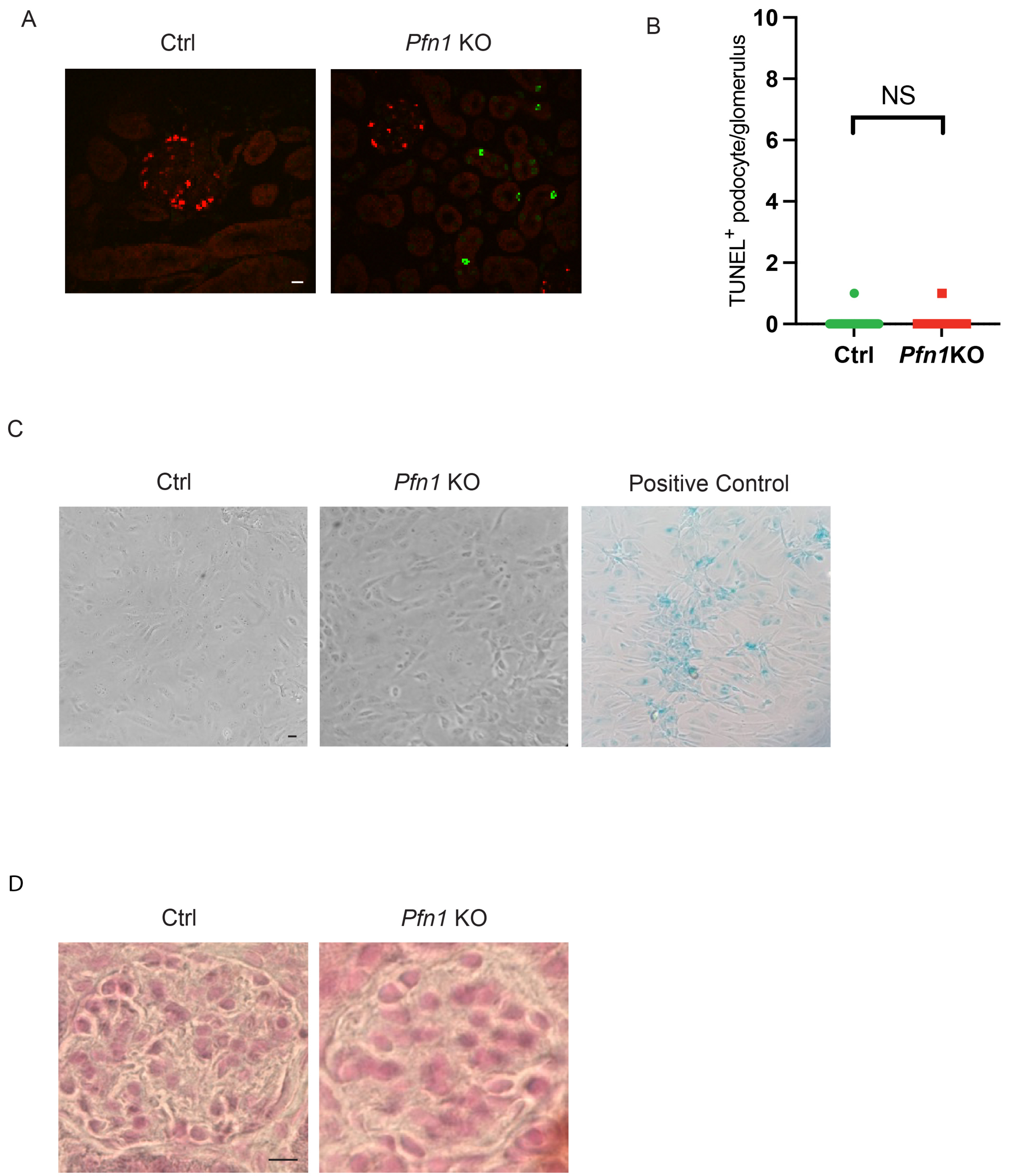

Supplemental Figure 4. Podocyte apoptosis or cellular senescence is not observed in *Pfn1* KO mice. (A) Representative images of TUNEL assay (green, representing apoptotic cells) co-stained with WT1 (red) in glomeruli of Ctrl and *Pfn1* KO mice at 4 weeks of age. Scale bar: 20μm. **(B)** Quantification of TUNEL-positive podocytes of glomeruli in **A**. Total 100 podocytes from n=5 mice in each group. P >0.05 vs Ctrl, no significance (NS). Statistically analyzed via a 2-tailed t test. **(C)** Representative images of senescence-associated β-galactosidase (SA-β-gal) activity, a in primary podocytes isolated from Ctrl and *Pfn1* KO mice at P7. Blue coloration in positive control represents cellular senescence. Scale bar: 10μm. **(D)** Representative images of SA-β-gal activity in glomeruli from Ctrl and *Pfn1* KO mice at 4 weeks of age. Scale bar: 20μm.

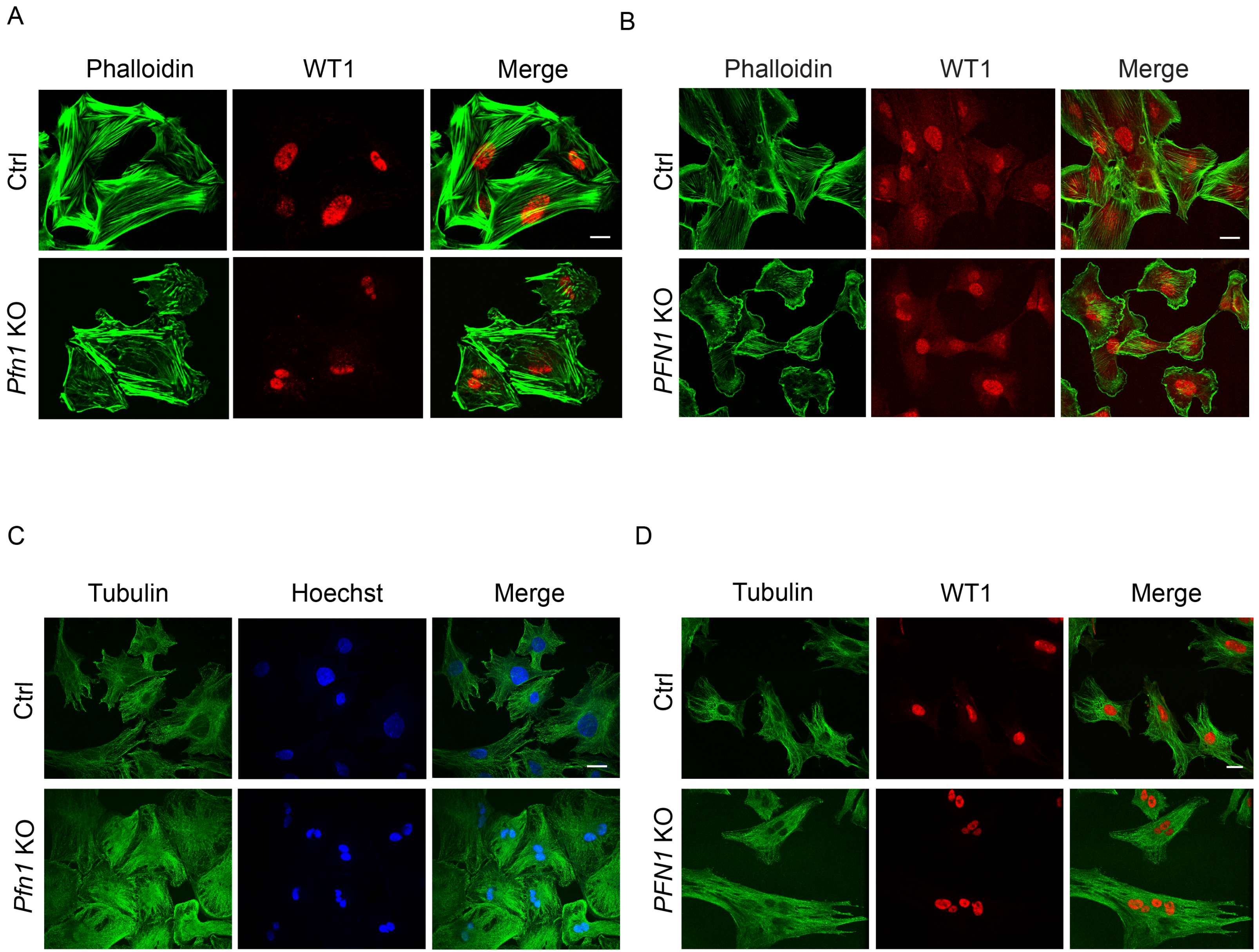

Supplemental Figure 5. Cytoskeleton remains relatively intact in mouse *Pfn1* KO and human *PFN1* KO podocytes in vitro. (A-B) Representative immunofluorescence images of phalloidin (green) and WT1(red) in mouse Ctrl and *Pfn1* KO primary podocytes (age P7) in **A**, or in human Ctrl and *PFN1* KO podocytes in **B**. **(C-D)** Representative immunofluorescence images of tubulin (green) and hoechst (blue) in mouse Ctrl and *Pfn1* KO primary podocytes in **C**, or tubulin (green) and WT1(red) in human Ctrl and *PFN1* KO podocytes in **D**. Scale bar: 20µm.

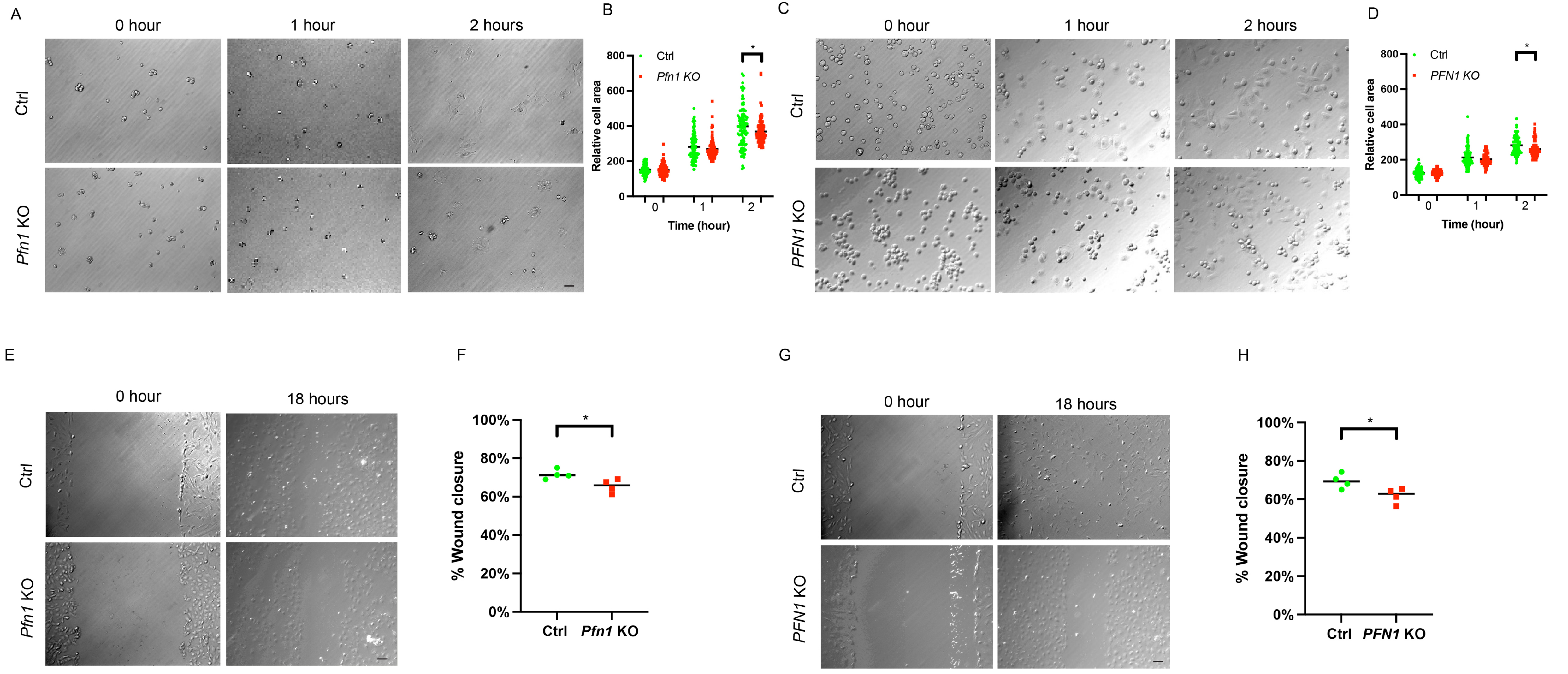

Supplemental Figure 6. Impaired spreading and wound healing migration in mouse *Pfn1* KO and human *PFN1* KO podocytes. (A) Representative images of mouse Ctrl and *Pfn1* KO podocytes at times 0, 1 hour, and 2 hours. **(B)** Quantification of mouse podocyte spreading assay in **A**. Total 100 cells in 4 independent experiments. **(C)** Representative images of human Ctrl and *PFN1* KO podocytes at times 0, 1 hour, and 2 hours. **(D)** Quantification of human podocyte spreading assay in **C**. Total 100 cells in 4 independent experiments. **(E)** Representative images of mouse Ctrl and *Pfn1* KO podocytes at time 0 following wound initiation, and 18 hours after wound healing migration. **(F)** Quantification of mouse podocyte migration assay in **E**. n=4 independent experiments. **(G)** Representative images of human Ctrl and *PFN1* KO podocytes at time 0 following wound initiation, and 18 hours after wound healing. **(H)** Quantification of human podocyte migration assay in **G**. n=4 independent experiments. Scale bar: 20μm. **P*<0.05 vs Ctrl. Statistically analyzed via a 2-tailed *t* test.

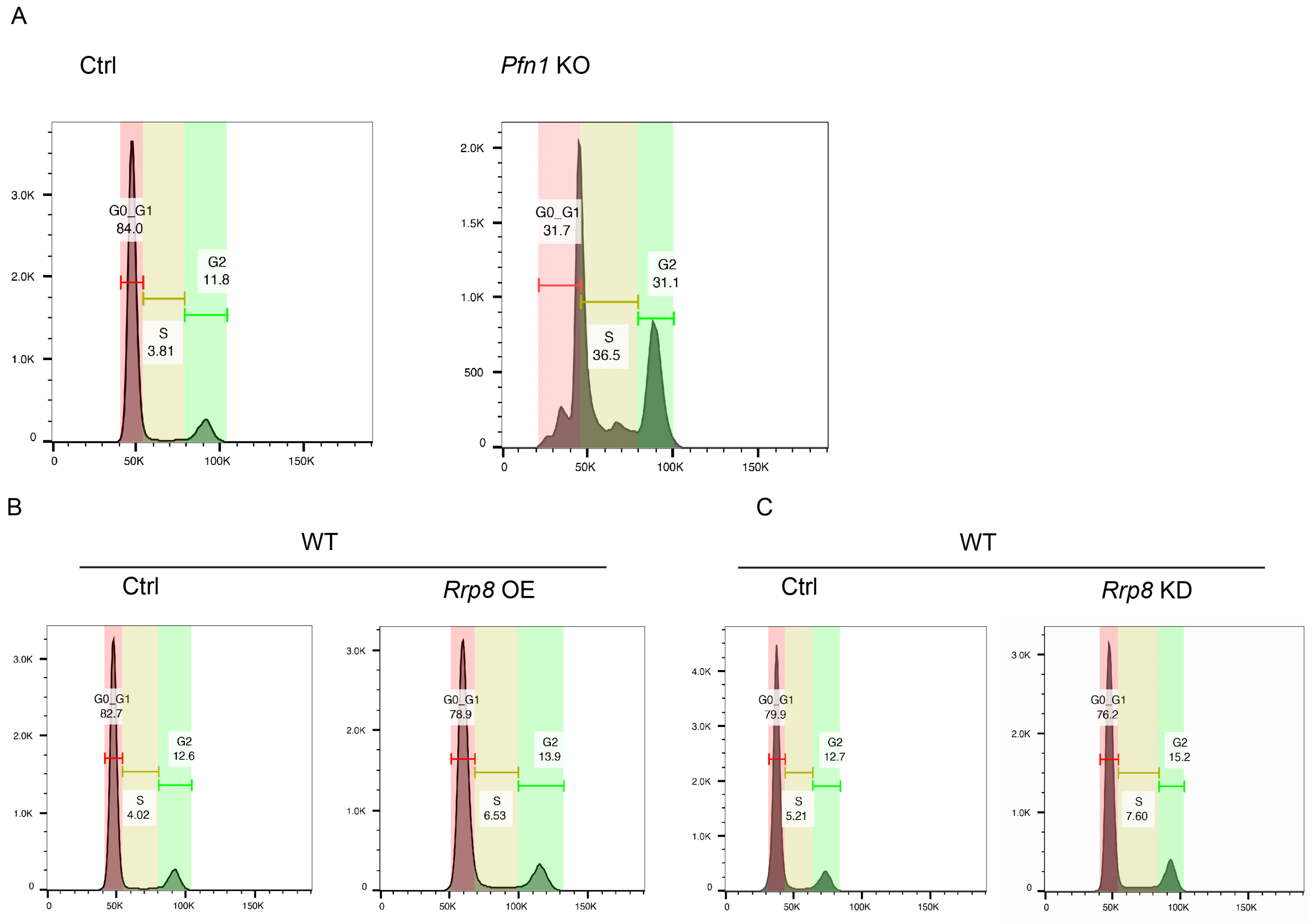

Supplemental Figure 7. Flow cytometric analysis of the cell cycle phases in podocytes in vitro. (A) Cell cycle phases in primary podocytes isolated from *Pfn1* KO and Ctrl mice at P7. **(B-C)** Cell cycle phases in primary podocytes isolated from WT mice after transduction with mouse *Rrp8* lentiviral activation particles (over-expression [OE] of *Rrp8*) **(B)** or with *Rrp8* shRNA lentiviral particles (knockdown [KD] of *Rrp8*) **(C)** compared to their respective Ctrls.

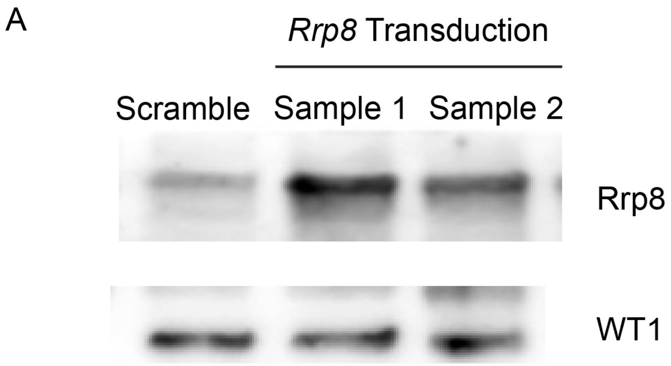

Supplemental Figure 8. The efficiency of mouse Rrp8 expression in *Pfn1* KO primary podocytes. **(A)** Immunoblot images of Rrp8 expression in primary podocytes isolated from *Pfn1* KO mice (age P7) after transduction with control lentiviral activation particles (scramble) or mouse *Rrp8* lentiviral activation particles (sample1 and sample2), along with WT1 blotting as the loading control.

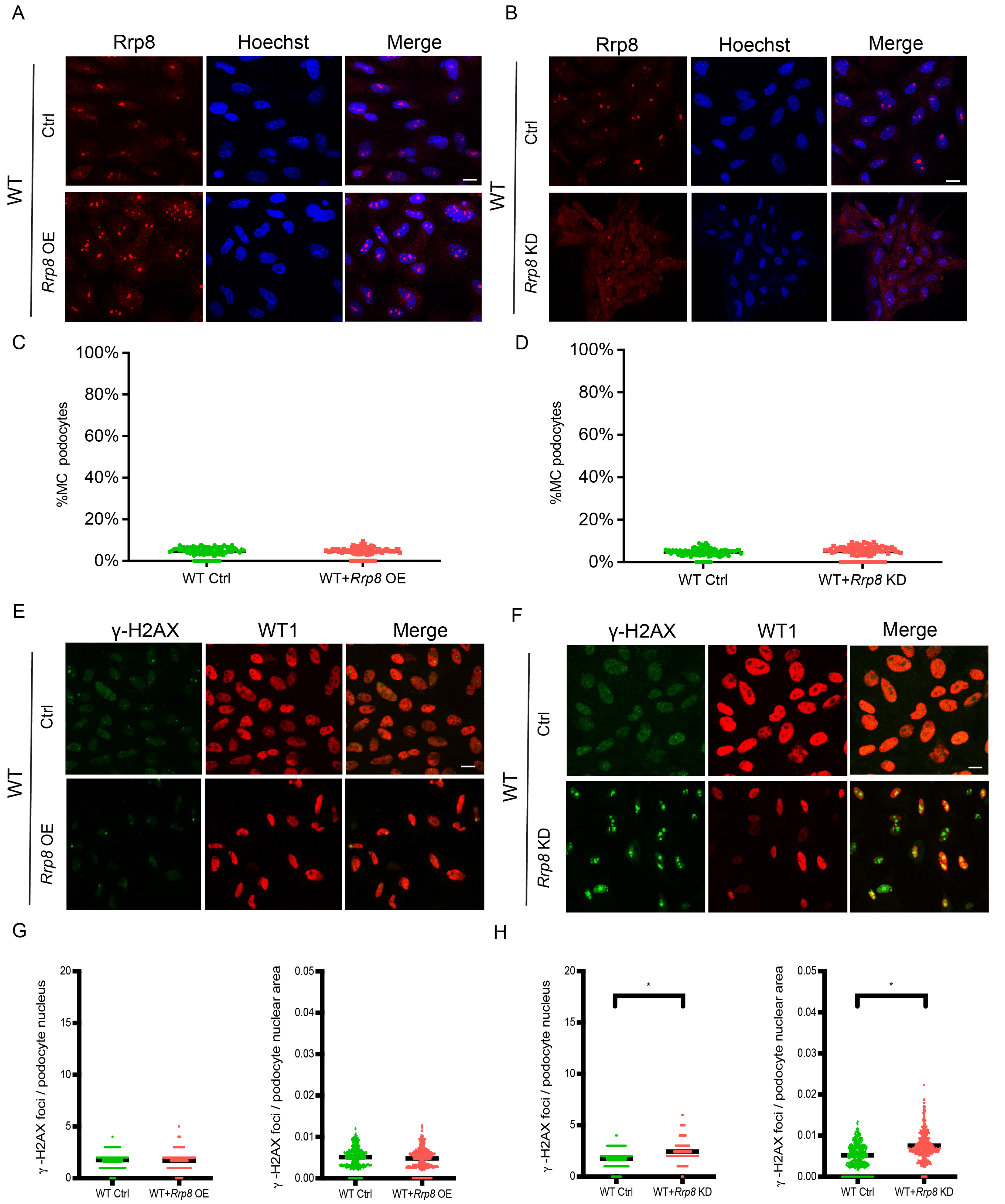

Supplemental Figure 9. In wild type (WT) podocytes, Rrp8 displays no effect on the mitotic catastrophe, and knockdown of *Rrp8* increases the DNA damage. (A-B) Representative immunofluorescence images of mouse WT podocytes with *Rrp8* over-expression (OE) **(A)** or knockdown (KD) **(B)** , stained with Rrp8 (red) and Hoechst (blue). **(C)** Quantification of the percentage of MC podocytes per field of view in **A**. **(D)** Quantification of the percentage of MC podocytes per field of view in **B**. Total 100 fields of view in 5 independent experiments. **(E-F)** Representative immunofluorescence images of WT podocytes with *Rrp8* OE **(E)** or KD **(F)**, stained with γH2AX (green) and WT1 (red). **(G)** Quantification of γH2AX foci per podocyte nucleus (left) and per podocyte nuclear area (right) in **E**. **(H)** Quantification of γH2AX foci per podocyte nucleus (left) and per podocyte nuclear area (right) in **F**. Total 400 cells in 5 independent experiments.*$P<0.05$ vs Ctrl.Statistically analyzed via a 2-tailed *t* test. Scale bar: 20μm.

Supplemental Table 1

Primers of Real-time PCR.

Mouse PCR Primers for genotyping

Gene Name	Sequences (5'-3')
*Pfn1*_FW	5′-TGGAGCGGATCCAGCGAAGG -3′
*Pfn1*_RV1	5′-GTCCCCAGCAGTCGGGACG-3′
*Pfn1*_RV2	5′-TGGAGCGGATCCAGCGAAGG -3′
*Pfn1*_RV3	5′-GGACACCAACCTCAGCTGGC-3′
*Pod-Cre*_FW	5′-ACAGCTCCACCAAGACACAG -3′;
*Pod-Cre*_RV	5′-TCCGGTTATTCAACTTGCACC-3′
*Dtr*_FW	5′-CACTGGATCTACGGACCAGC-3′
*Dtr*_RV	5′-CGATTTTCCACTGGGAGGCT-3′
*R26Fucci2aR*_FW	5′-CAAAGTCGCTCTGAGTTGTTATCAG-3′
*R26Fucci2aR*_RV1	5′-GGAGCGGGAGAAATGGATATGAAG-3′
*R26Fucci2aR*_RV2	5′-TGGCGGCCGCTCGAGATGAATC-3′
*TRAP(L10a)*_FW	5′-AGTGCTTCAGCCGCTACC -3′
*TRAP(L10a)*_RV	5′-CTTGCCAGCCTTGTTTAGG-3′

Mouse qPCR Primers

Gene Name	Sequences (5'-3')
*Kat2b*_FW	CTGCTTCCTGCGGGC
*Kat2b*_RV	GCATTTACAGGACTCCTCTGC
*Dicer1*_FW	CGATGGTTCTGGAAGGCCC
*Dicer1*_RV	GAAGCAGGGGTCATGAGCTG
*Rrp8*_FW	ATGTCGTCCATAGCAGCGTC
*Rrp8*_RV	GCTTCCAGAGCCCGTAATGT
*Pfn1*_FW	CGGGTGGAACGCCTACATCG
*Pfn1*_RV	TGACCGGTCTTTGCCTACCAG
*Gapdh*_RV	TCACCACCATGGAGAAGGC
*Gapdh*_RV	GCTAAGCAGTTGGTGGTGCA

Human qPCR primers

Gene Name	Sequences (5'-3')
*PFN1*_FW	CTCACTGCTGCAGGATGGGGAAT
*PFN1*_RV	AAAGCTGTGGGGAGCGGTGAA
*GAPDH*_RV	CCACTCCTCCACCTTTGAC
*GAPDH*_RV	ACCCTGTTGCTGTAGCCA

Supplemental Table 4. Clinical Characteristics of Patients with Different Proteinuric Kidney Disease

Proteinuric Chronic Kidney Disease		Age，years	Male:Famale	SBP, mmHg	DBP,mmHg	TP, g/L	ALB, g/L	TCHO,mmol/L	TG, mmol/L	Scr, μmol/L
FSGS	Non-MC Podocytes	39±16	9:8	129±11	85±10	57.5±10.3	33.3±8.7	6.30±1.92	2.52±1.47	137.10±182.83
	MC Podocytes	31±10	1:2	124±9	87±9	52.8±16.1	28.3±12.6	8.06±5.68	3.80±2.91	55.83±24.76
	P value	0.422	1.00	0.765	0.616	0.616	0.397	0.648	0.358	0.616
DKD	Non-MC Podocytes	51±12	15:8	147±14	87±9	51.8±14.1	28.2±7.1	5.41±1.36	2.20±1.33	104.48±62.33
	MC Podocytes	54±14	3:0	148±11	87±12	65.3±11.9	36.1±11.9	4.13±1.56	1.40±0.62	80.66±44.51
	P value	0.753	0.529	0.857	0.975	0.128	0.104	0.142	0.275	0.541
pMN	Non-MC Podocytes	48±14	77:52	127±17	80±12	49.9±9.2	28.5±24.4	12.38±58.67	2.68±1.62	60.42±59.61
	MC Podocytes	45±14	10:6	124±27	85±16	48.2±8.6	25.7±7.8	8.36±2.59	2.54±1.05	61.08±30.90
	P value	0.480	0.829	0.419	0.303	0.394	0.540	0.127	0.840	0.892
LN	Non-MC Podocytes	34±12	4:26	127±18	84±13	55.4±14.6	28.0±7.1	5.41±1.75	1.88±0.72	74.62±49.20
	MC Podocytes	29±8	0:3	132±4	93±18	40.4±8.3	19.4±5.2	7.42±0.95	2.45±0.82	38.31±3.63
	P value	0.483	1.00	0.629	0.298	0.045	0.050	0.061	0.235	0.149

IgAN	Non-MC Podocytes	36±13	54:49	124±15	81±10	63.7±12.1	37.0±8.1	4.99±1.67	1.90±1.87	83.71±77.31
	MC Podocytes	35±11	3:4	136±26	87±12	59.2±10.1	35.2±8.4	5.77±1.99	2.42±1.45	72.32±13.44
	P value	0.995	0.447	0.378	0.126	0.192	0.528	0.223	0.072	0.565

Abbreviation: MC: Mitotic catastrophe; Non-MC: non-mitotic catastrophe; FSGS: Focal segmental glomerulosclerosis; DKD: Diabetic kidney disease. pMN: Primary membranous nephropathy; LN: Lupus nephritis. IgAN: Immunoglobulin A nephropathy; SBP: Systolic blood pressure; DBP: Diastolic blood pressure; TP: Total protein; ALB: Albumin; TCHO: Total cholesterol; TG: Triglyceride； Scr: Serum creatinine

Supplemental Table 5 Image J Macro File

```
var tolerance = 30;

run("Split Channels");
waitForUser("Close blue channel and select red channel");

originalImage = getTitle();
run("8-bit");
run("Overlay Options...", "stroke=red width=1 fill=none set");
run("Median...", "radius=6");
run("Subtract Background...", "rolling=125 sliding");
run("Auto Threshold", "method=Default white");
roiManager("Reset");
run("Analyze Particles...", "pixel exclude clear add");

waitForUser("Select green channel");
run("8-bit");

run("Gaussian Blur...", "sigma=1");
run("Subtract Background...", "rolling=25");

for(i=0; i<roiManager("count"); i++) {
        roiManager("select", i);
        run("Find Maxima...", "noise="+tolerance+" output=[Count]");
        run("Find Maxima...", "noise="+tolerance+" output=[Point
Selection]");
        run("Add Selection...");
}

waitForUser("Please copy results before continuing.")

for(i=0; i<roiManager("count"); i++) {
        roiManager("select", i);
        run("Measure");
}
```
