# Supplementary figures and images for: Profilin1 is required for prevention of mitotic catastrophe in murine and human glomerular diseases
Source: J Clin Invest. 2023 Dec 15;133(24):e171237. doi: 10.1172/JCI171237 (PMC10721156; doi:10.1172/JCI171237)

Figure 1A

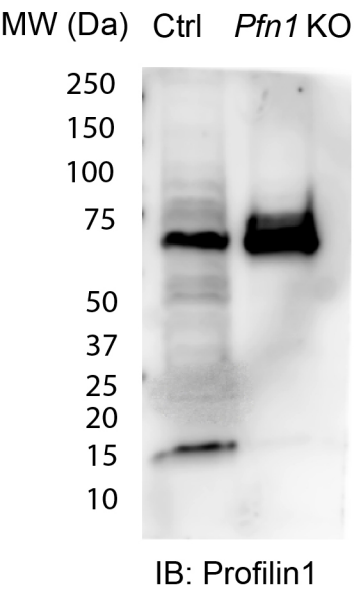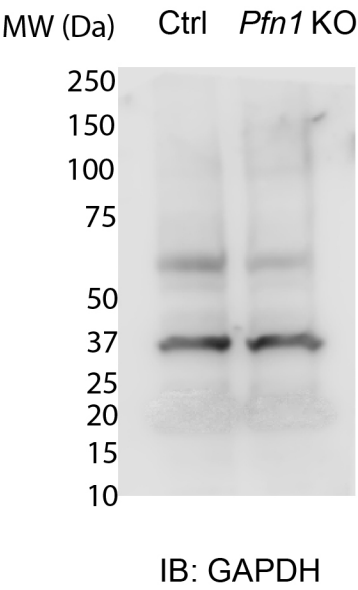

Figure 4A

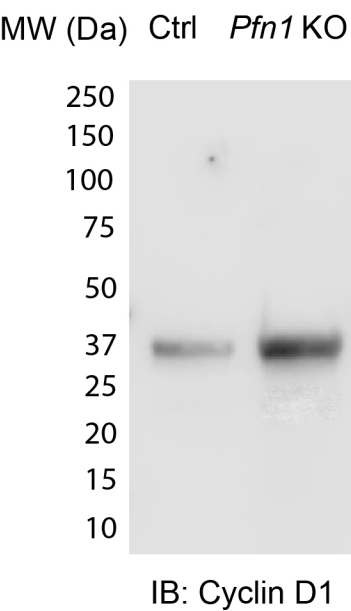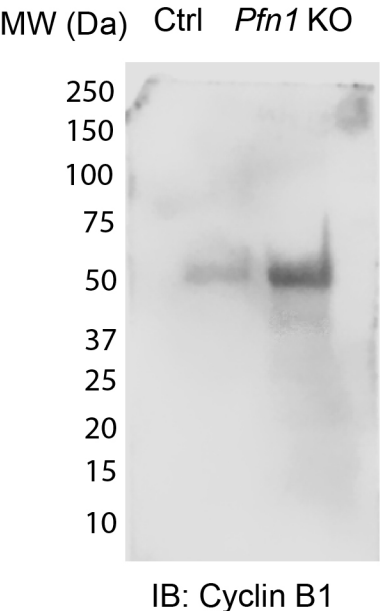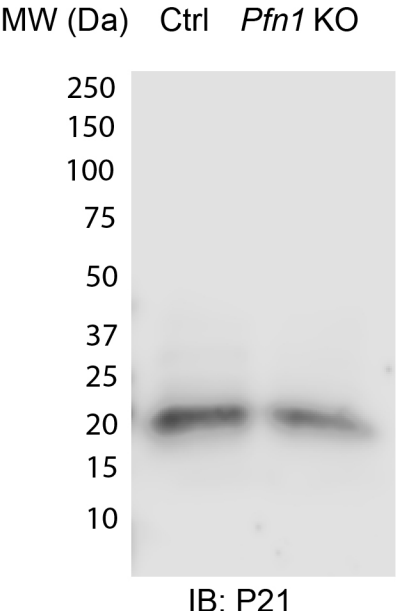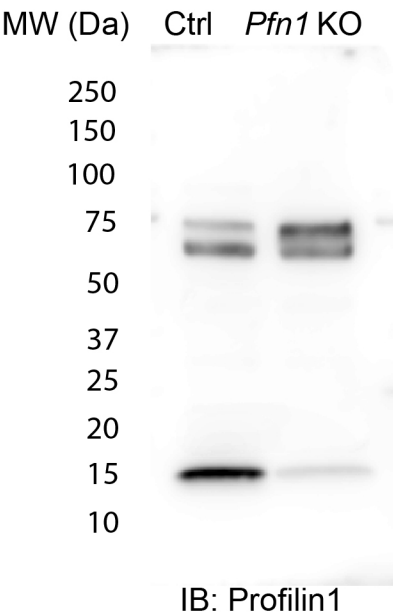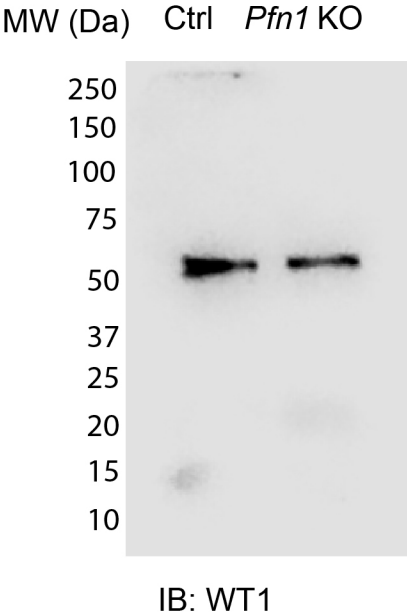

Supplementary Figure 2B

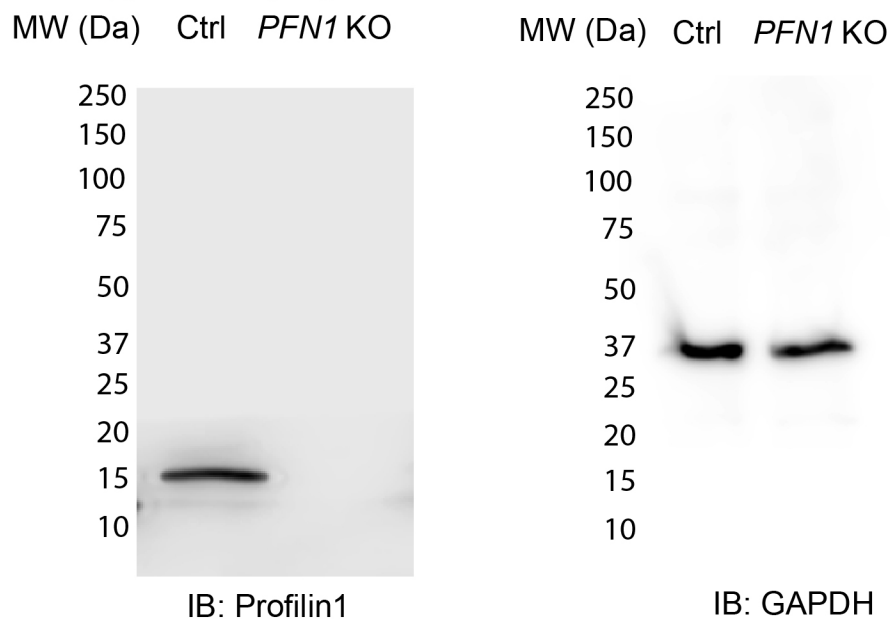

Supplementary Figure 2G

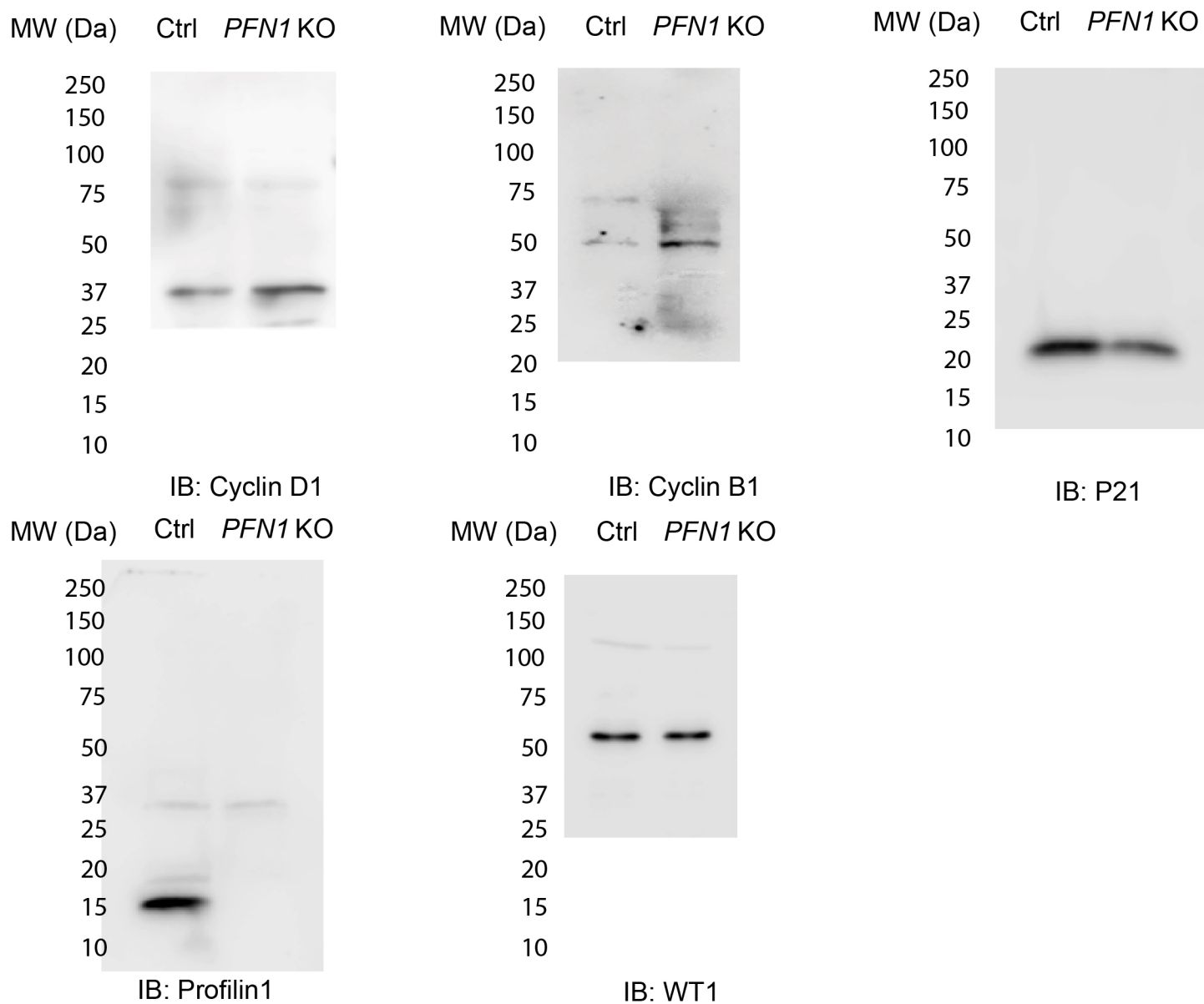

Supplementary Figure 5A

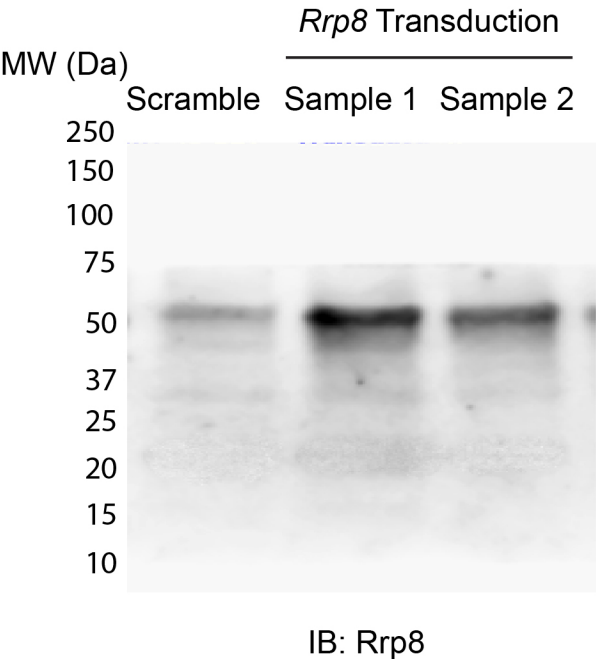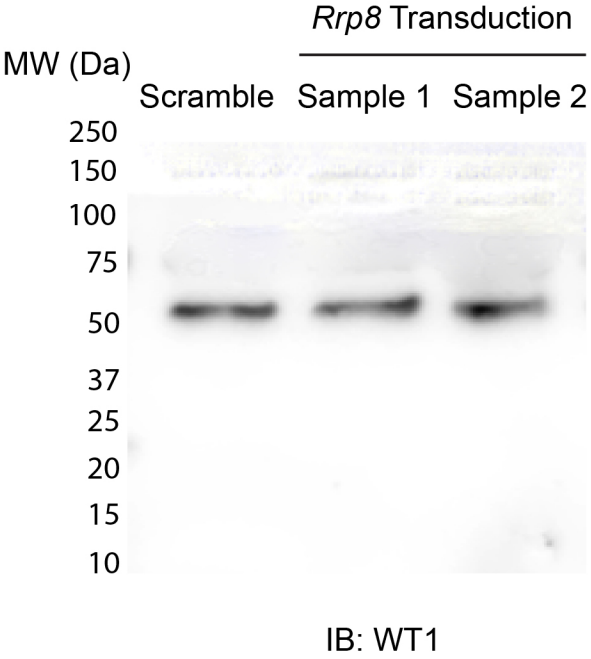

Supplement: Unedited blot and gel images [file jci-133-171237-s213.pdf]
